# A Systematic Review of Recent Developments in Wound Healing and Skin Regeneration Properties of Plant-Extract-Based Hydrogels for Skin Delivery: A Focus on Asteraceae and Lamiaceae Families

**DOI:** 10.3390/pharmaceutics18091047

**Published:** 2026-08-23

**Authors:** Monika Michalak

**Affiliations:** Department of Pharmaceutical Sciences, Collegium Medicum, Jan Kochanowski University, IX Wieków Kielc 19, 25-369 Kielce, Poland; monika.michalak@ujk.edu.pl

**Keywords:** wound healing, skin regeneration, plant extracts, Asteraceae, Lamiaceae, hydrogels, topical delivery

## Abstract

**Background:** Plants have been traditionally used for centuries to treat wounds and, over time, have been tested for their healing properties. There is a constant search for new natural resources that could be used to develop various topical wound care products. **Methods:** A comprehensive search of the literature was conducted in PubMed/MEDLINE, Scopus, and Web of Science databases (2022–2026) in accordance with the PRISMA 2020 guidelines. Included studies focused on current in vitro and in vivo research on hydrogels containing plant extracts from the Asteraceae and Lamiaceae families and their potential application in wound healing and skin regeneration. **Results:** An analysis of 24 included studies confirms that both families include interesting and valuable plants with antioxidant, antimicrobial and anti-inflammatory properties; these plants also influence collagen deposition, fibroblast proliferation, and epithelialization, and reduce the risk of infection, thereby contributing to faster wound healing. The most frequently tested phytoextract in this respect was *Calendula officinalis* (Asteraceae) incorporated into a hydrogel. A variety of materials, including natural, semi-synthetic, and synthetic polymers, as well as hybrid matrix, but also diverse formulation strategies, from simple solutions to more advanced and modern methods, have been used to produce hydrogels. **Conclusions:** This systematic review summarizes the current evidence, highlights directions and possibilities for the use of phytoextract-based hydrogels, and discusses limitations and future perspectives in the development of effective externally applied formulations to support wound healing.

## 1. Introduction

Medicinal plants have been used for thousands of years to prevent and treat many skin ailments, including wounds, cuts, and burns. Contemporary research draws upon this knowledge, focusing on scientifically confirming the efficacy of plants known and previously used, or on discovering new species and bioactive compounds with healing and regenerative properties [1]. Many scientific papers are devoted to researching and testing extracts for various pharmacological purposes, including mechanisms of action relevant to wound healing, to benefit from the vast natural chemical diversity. Research suggests that it is worth considering the inclusion of phytotherapeutic drugs in the treatment of difficult-to-heal wounds, especially for persistent or treatment-resistant skin ulcers [2]. Data from the literature indicate that among the plants from different families that can potentially be used in wound-healing therapy, due to their widespread availability, low number of undesirable effects and their overall effectiveness, such species as *Achillea millefolium*, *Aloe vera*, *Althaea officinalis*, *Calendula officinalis*, *Matricaria chamomilla*, *Curcuma longa*, *Betula pendula*, *Equisetum arvense*, *Eucalyptus globulus*, *Camellia sinensis*, *Punica granatum*, *Plantago lanceolata*, *Glycyrrhiza glabra*, *Epilobium angustifolium*, *Nigella sativa*, *Pinus sylvestris*, *Centella asiatica*, *Quercus robur*, *Epilobium angustifolium*, *Cumin carvi*, *Salix koreensis*, *Virola oleifera*, can be mentioned, among others. To date, various plant species, as well as extracts, essential oils, hydrolats, oils, juices, honey, fermented products, mucilages, and plant-derived bioactive compounds, have been investigated for use either independently or as an adjunct to therapy [2,3,4]. These plant-derived raw materials can be used in a variety of dosage forms for topical application to wounds, ranging from conventional dosage forms (e.g., creams, ointments, emulsions, solutions) to novel drug delivery systems (e.g., specialized dressings, hydrogels, microspheres, nanoparticles, liposomes, nanoemulsions, microemulsions, phytosomes, nanofibers) that support wound healing [4].

Although numerous studies have focused on hydrogels containing plant extracts from species belonging to various botanical families, with results confirming their promising impact on wound healing, the available data are scattered across individual publications and are difficult to compare directly. To date, no systematic review of the literature has been conducted focusing on hydrogels containing extracts from plant families such as Asteraceae and Lamiaceae. Therefore, it is justified to conduct a review that will allow for a comprehensive presentation of available scientific evidence, a thorough summary of current knowledge, and the identification of directions for future research. This systematic review aims to critically synthesize and evaluate the current evidence (2022–2026) in the field of hydrogels containing plant extracts from species belonging to the Asteraceae and Lamiaceae families, as well as their potential applications in supporting skin regeneration and accelerating wound repair and healing.

## 2. Bioactivity of Plants in Wound Healing and Skin Regeneration

**Figure 1:**
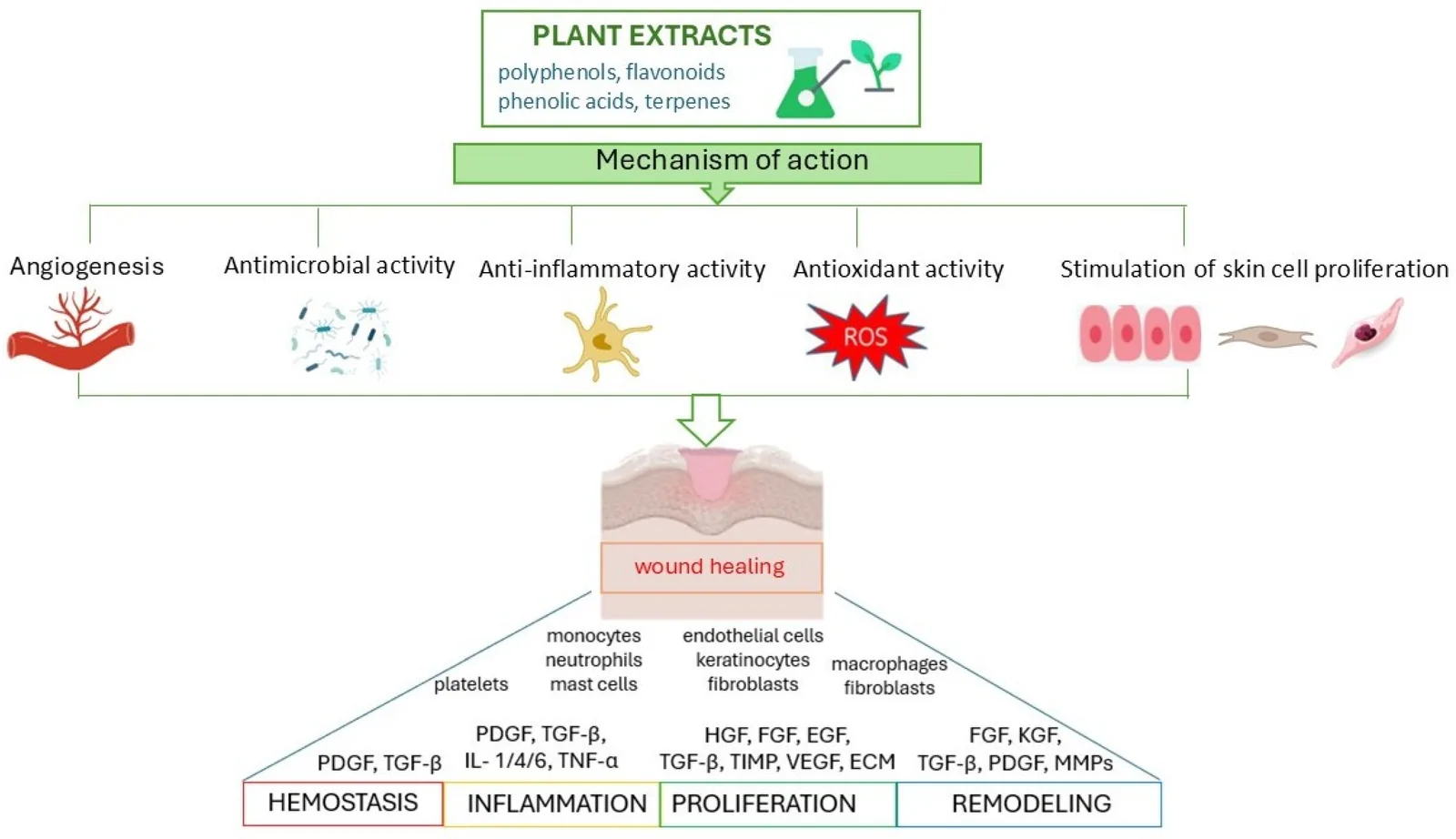
The importance of plants in wound healing.

Data from the literature confirm that plant-derived raw materials, due to their content of bioactive substances belonging to various groups of chemical compounds (e.g., flavonoids, phenolic acids, terpenes) with multifaceted effects (including antimicrobial, anti-inflammatory, antioxidant activity, as well as the stimulation of skin cell proliferation and angiogenesis), can act at various stages of the wound-healing process (Figure 1) [1,2,3,4].

### 2.1. Asteraceae and Lamiaceae Families Characterization

Asteraceae, known as the sunflower family, and Lamiaceae, also known as the mint family, are among the largest flowering plant families in the world. They are found on almost every continent and grow in every type of habitat due to their easy ecological adaptation and cosmopolitan distribution. Many members of the Asteraceae (e.g., *Calendula officinalis*, *Arnica montana*, *Matricaria chamomilla*, *Silybum marianum*, *Tanacetum parthenium*, *Achillea millefolium*, *Carthamus tinctorius*, *Artemisia absinthium*, *Cynara cardunculus*, *Grindelia robusta*) and the Lamiaceae (e.g., *Melissa officinalis*, *Mentha piperita*, *Rosmarinus officinalis*, *Marrubium vulgare*, *Lavandula officinalis*, *Hyssopus officinalis*, *Salvia officinalis*, *Thymus vulgaris*, *Origanum vulgare*, *Scutellaria baicalensis*) families have therapeutic use and a long history in traditional medicine. Herbs from the Asteraceae and Lamiaceae families are medicinal plants with properties that are widely used in the pharmaceutical industry due to the various effective compounds they contain [5,6,7,8,9,10,11,12,13]. An analysis of the scientific literature indicates the significant potential of plants from the Asteraceae and Lamiaceae families in wound healing and skin regeneration, as both families are rich sources of active compounds such as polyphenols, flavonoids, terpene compounds, saponins, and essential oils, which exhibit multifaceted biological effects [1,6,14]. It is worth emphasizing that the effectiveness of plant extracts often results from the synergistic action of many bioactive compounds that can affect many pathways simultaneously, which may lead to a more comprehensive and stronger therapeutic effect than single, isolated compounds. Among the most important phytochemicals found in Asteraceae and Lamiaceae species with scientifically proven wound-healing efficacy are volatile terpenes (e.g., 1,8-cineole, linalool, thymol, pinene, camphene, camphor, carvacrol), flavonoids (e.g., kaempferol, luteolin, quercetin, rutin, apigenin, and its derivatives), phenolic acids (e.g., rosmarinic acid, caffeic acid, chlorogenic acid, *p*-coumaric acid, ferulic acid, caffeoylquinic acid), and di- and triterpenoids (e.g., ursolic acid, marrubin, carnosol, oleanolic acid, carnosic acid) [6,8,9]. These bioactive compounds exhibit multifaceted effects, including antioxidant, anti-inflammatory, and antimicrobial properties, as well as the ability to stimulate cell proliferation and angiogenesis, which are essential for wound healing [9,15,16]. Plants belonging to the Asteraceae and Lamiaceae families, which contain such bioactive compounds, represent a promising source for the development of new, effective natural preparations that support skin regeneration and healing processes.

### 2.2. Plant Mechanisms of Action Relevant to the Healing Process and Methods for Assessing Bioactivity

The use of multipurpose plant raw materials is particularly important in complex wounds such as burns, chronic wounds, and diabetic foot ulcers, as inappropriate treatment can excessively prolong the healing process. This is because these wounds are accompanied by local wound hypoxia and chronic inflammation, which, in turn, increase the wound’s susceptibility to bacterial infection, further delaying and hindering proper healing.

The process of skin regeneration and healing consists of stages such as hemostasis, inflammation, proliferation, and remodeling. It involves interactions among multiple cell types, including endothelial cells, inflammatory cells, keratinocytes, and fibroblasts [3]. Skin damage triggers the initial healing process, aimed at halting blood loss and preventing pathogen entry, which is characterized by vasoconstriction, platelet aggregation, and fibrin formation. This phase is characterized by heightened platelet activity, involving the release of coagulation-inducing substances (e.g., fibrinogen, fibronectin, and thrombospondin) and growth factors (platelet-derived growth factor-PDGF and transforming growth factors alpha and beta-TGF-α, TGF-β) that stimulate the chemotaxis and proliferation of monocytes and macrophages, and, subsequently, fibroblasts. The inflammatory phase is initiated by the sequential migration of neutrophils (essential for the removal of pathogens and cellular debris), macrophages (which secrete a range of proinflammatory cytokines, e.g., TNF-α and IL-1), and lymphocytes (which regulate the inflammatory response and facilitate the transition to the proliferative phase) into the wound, driven by signals transmitted by PDGF, TGF-β, and FGF (fibroblast growth factor). The proliferation phase is characterized by epithelialization, angiogenesis, and extracellular matrix (ECM) biosynthesis. Mediators and growth factors produced by fibroblasts (IGF-1 (insulin-like growth factor 1), PDGF, EGF (epidermal growth factor), TGF-β, bFGF (basic fibroblast growth factor)), keratinocytes (TGF-α, TGF-β, and KDAF (keratinocyte-derived autocrine factor)), and endothelial cells (VEGF (vascular endothelial growth factor), bFGF, and PDGF) are responsible for modulating and stimulating these processes. The final stage of wound healing is remodeling, during which maturation and epithelial tissue formation occur, ultimately leading to the formation of scar tissue. The entire process is driven by numerous growth factors and cells, with the individual phases of wound healing occurring partly in parallel, ensuring continuous remodeling [17,18,19].

The efficacy of plants in the wound-healing process stems from various mechanisms, including antioxidant, anti-inflammatory, and antimicrobial activities, as well as the direct stimulation of regenerative processes such as fibroblast and keratinocyte proliferation and angiogenesis [1,20]. Data from the literature confirm that plant-derived raw materials can act at various stages of the wound-healing process [21].

#### 2.2.1. Antioxidant Activity

Various theories explaining the mechanism of impaired wound healing at the molecular level are linked by the hypothesis of a pro- and antioxidant imbalance in the wound, resulting in excessive and uncontrolled formation of reactive oxygen species (ROS) and, consequently, oxidative stress. It is a major factor in cell damage (proteins, lipids, nucleic acids) and delays wound healing. Data from the literature indicate the important role of antioxidants in improving wound healing, especially given the high content of free radicals in chronic ulcers, which impairs all phases of wound healing. It is worth noting that ROS plays a key role in preparing the proper wound-healing response and mediates such cellular processes as growth, proliferation, differentiation, and apoptosis; hence, the basis of new therapies is antioxidants and antioxidant dressings that regulate the balance between low and high ROS levels [1,22]. The antioxidant properties of plant bioactive compounds result from various mechanisms of action, including binding free radicals (stabilizing or delocalizing unpaired electrons, interrupting free radical chain reactions, reducing properties (donating an electron or hydrogen atom), or chelating metal cations (e.g., Cu^2+^, Fe^2+^) from enzymes that catalyze oxidation reactions. Plant antioxidants also increase fibroblast activity and collagen synthesis, which are essential for tissue regeneration [1,23]. The following tests are used to assess the antioxidant properties of plants and their components: radical scavenging assays (e.g., DPPH (2,2′-diphenyl-1-picrylhydrazyl), ABTS (2,2′-azinobis-(3-ethylbenzothiazoline-6-sulfonic acid)), lipid peroxidation assays (e.g., TBARS (thiobarbituric acid reactive substances), FRAP (the ferric antioxidant power reduction), CUPRAC (cupric ion reducing antioxidant capacity), and cell- and enzyme-based assays such as cellular antioxidant activity assay (e.g., intracellular levels of reactive oxygen species (ROS)) and inhibition of antioxidant enzymes (e.g., superoxide dismutase (SOD), catalase (CAT)), among others [10,23].

#### 2.2.2. Anti-Inflammatory Activity

Plant-derived anti-inflammatory substances play a significant role in the wound-healing process. Inflammation is a crucial phase in the entire healing process, but excessive production of some inflammatory mediators and prolonged inflammation can delay wound healing. Bioactive plant compounds modulate the inflammatory response, preventing chronic inflammation and preparing the wound for tissue repair. Plant-derived materials have been shown to inhibit the formation of proinflammatory cytokines and eicosanoids, preventing the inflammatory cascade [1]. The assessment of the anti-inflammatory effects of plants may range from quick and low-cost in vitro laboratory tests to advanced in vivo studies. In vitro cell-free or cell culture (e.g., macrophage RAW 264.7 cell lines) experiments measure inflammatory biomarkers, including anti-inflammatory enzymes and proteins such as cyclooxygenase (COX), lipoxygenase (LOX), hyaluronidase (HAase), as well as the production of proinflammatory cytokines such as tumor necrosis factor (TNF), interleukins (IL-1β, IL-6, IL-8) and nitric oxide (NO) synthesized by inducible NO synthases (iNOS). More challenging is measuring such intracellular inflammatory transcription factors as nuclear factor (NF)-κB, nuclear factor of activated T cells, and activated protein (AP)−1 [24,25]. In vivo animal models (rats, mice, rabbits), simulating complex inflammatory responses in living organisms, allow for the evaluation of the actual therapeutic effects of plant extracts on wound healing. Such studies typically combine edema models (carrageenan-induced paw edema and croton-oil-induced ear edema), macroscopic wound measurements, and histopathology to assess the therapy’s effectiveness in reducing edema and promoting tissue repair during the standard wound-healing period [26,27].

#### 2.2.3. Antimicrobial Activity

Plants with antimicrobial properties constitute an important group of raw materials that support the healing process, significantly contributing to wound contraction and accelerating epithelialization [3]. Because infection can be a significant cause of delayed wound healing, an important approach in the treatment of chronic wounds is the use of topical antimicrobials, which are gaining importance compared to the use of systemic antibiotics due to increased resistance resulting from the overuse of antimicrobials [4]. Topically applied herbal products and bioactive plant compounds that exhibit a broad spectrum of antimicrobial activity (including destruction of microbial cell membranes, inhibition of nucleic acid synthesis and inhibition of biofilm formation) thereby protecting the wound from secondary infections and chronic inflammation, as well as reducing the risk of complications [1,20]. To determine antibacterial and antifungal activity, plants are tested against common pathogens causing wound infections (e.g., *Escherichia coli*, *Pseudomonas aeruginosa*, *Staphylococcus aureus*, methicillin-resistant *Staphylococcus aureus*, *Candida albicans*) using in vitro assays as agar well/disc diffusion assay to verify and compare efficacy and the broth dilution method to assess the minimum inhibitory concentration (MIC) and minimum bactericidal concentration (MBC)/minimum fungicidal concentration (MFC) [28,29].

#### 2.2.4. Proproliferative and Proregenerative Activity

Plants and their bioactive compounds can directly stimulate the proliferation of skin cells (fibroblasts, keratinocytes, endothelial cells), promote extracellular matrix (ECM) component production, and enhance collagen cross-linking, which is crucial for tissue repair and restoring skin integrity. In addition, some herbal agents support the formation of new capillaries (angiogenesis), which is essential for delivering oxygen and nutrients to regenerating tissues [1,20]. To confirm the proproliferative and proregenerative potential of plant extracts, various studies are conducted, ranging from simple cell proliferation tests to advanced molecular analyses, animal models, and clinical trials. In vitro studies, on, e.g., fibroblasts and keratinocytes at the cellular level, include cytotoxicity, proliferation, and cell viability assays (e.g., AB (alamar blue), NRU (neutral red uptake), MTT (3-(4,5-dimethyl thiazol-2-yl)-2,5-diphenyltetrazolium bromide) assay), cell differentiation and function assays (e.g., expression of TGF-β markers, collagen I/III), angiogenesis assays (e.g., tube formation assay (HUVEC (human umbilical vein endothelial cells)), VEGF (vascular endothelial growth factor) expression), and cell migration assays (e.g., wound-healing assay), while at the molecular level, studies analyze the impact on signaling pathways (e.g., MAPK/ERK (mitogen-activated protein kinase/extracellular signal-regulated kinase), PI3K/Akt (phosphoinositide 3-kinase/protein kinase B)) or the expression of genes (e.g., FGF-2 (basic fibroblast growth factor)) related to proliferation and regeneration [10,25,30,31,32]. In vivo studies include tests on animal models (most often rats/mice) (e.g., full-thickness excision, burn wound, diabetic models) allowing for tissue-level assessment of wound closure rate, angiogenesis, and collagen synthesis, as well as randomized clinical trials with patients (e.g., diabetic ulcers) allowing for the assessment of safety, healing time, and wound surface area reduction [26,33,34].

## 3. Hydrogel Scaffolds for Wound Healing and Skin Regeneration

**Figure 2:**
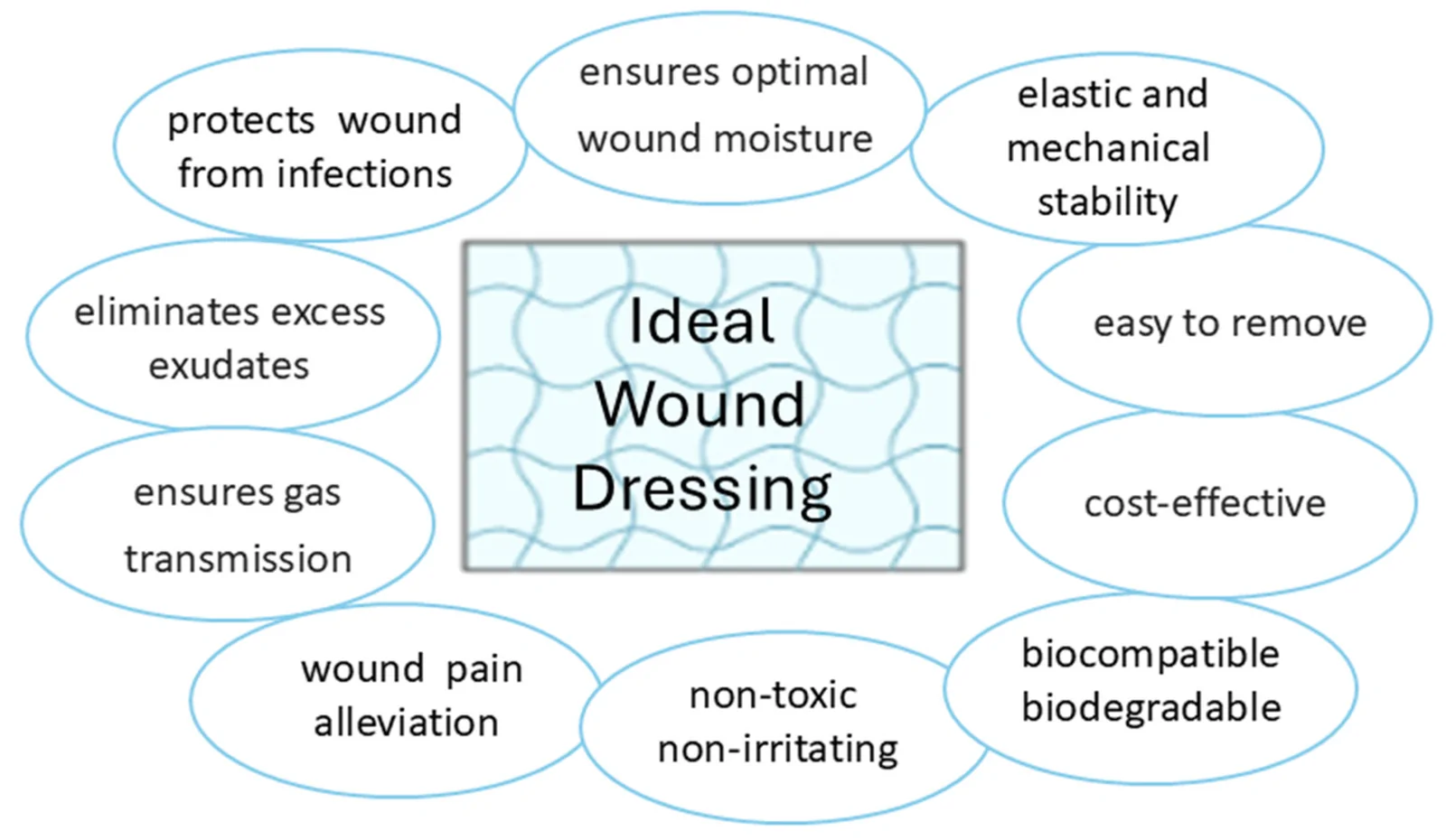
The features and properties of the ideal wound dressing.

Wound healing can be disrupted or inhibited by factors such as dryness, bacterial presence, pressure, necrotic tissue, and edema. Therefore, an effective wound dressing should act in multiple ways and fulfill functions such as protecting the wound from infection and creating a microenvironment conducive to tissue regeneration and skin wound healing. Furthermore, it should prevent overheating, ensure airflow, and be non-toxic, biocompatible and easy to remove without causing further damage [35,36,37,38] (Figure 2). Recent developments and research in biomaterials have demonstrated their significant role in the creation of new treatment methods. Medical textile materials, e.g., fibers, yarns, non-wovens, and electrospun nanofibrous materials, produced using advanced manufacturing techniques, are widely used in wound treatment. Advanced dressings also include foams, alginates, hydrogels, hydrocolloids, and hydrofiber dressings. Among those mentioned, hydrogels deserve attention; due to their close resemblance to natural tissues, they can be used in various fields of biomedicine, including regenerative medicine [1,35,36,38].

### 3.1. Classification and Characteristics of Hydrogels

**Figure 3:**
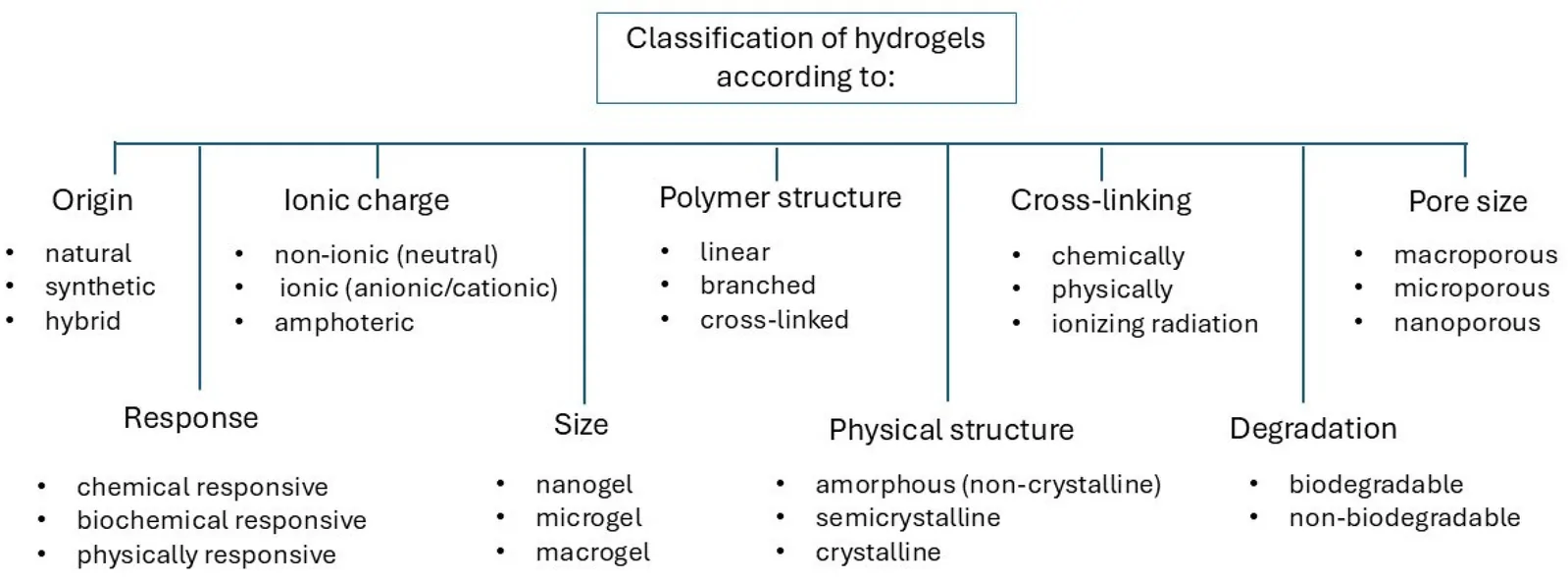
Classification of polymer gels.

Hydrogels are a versatile class of soft, semi-solid materials with a consistency intermediate between the liquid and solid states. These are systems in the form of a three-dimensional network of organic polymers. There are many classifications of polymer gels depending on their sources, polymeric composition, physical appearance or network electrical charge. Depending on the method of preparation, types of hydrogels such as homopolymer, copolymer, and multipolymer hydrogels, as well as interpenetrating network hydrogels, can be distinguished. Hydrogels are also classified based on their method of preparation, taking into account physical (hydrogen bonding, ionic interactions, metal-coordination interactions, and hydrophobic interactions) and chemical (conjugation reaction, free radical polymerization, enzymatic reaction) cross-linking. The cross-linking method determines the physicochemical properties and functions of the hydrogel dressing. Hydrogels can also be classified based on their degradability (capacity to break down via chemical reactions involving microorganisms or enzymes, resulting in their conversion into simpler compounds over a specific period), which depends on factors such as the degree of cross-linking, the polymer’s chemical composition and structure, and environmental conditions (e.g., temperature, pressure, and pH). Over the past few decades, hydrogels have evolved from conventional hydrogels to “smart” (stimuli-responsive) hydrogels capable of reversibly changing their properties (e.g., volume, degree of swelling, mechanical properties, and substance-release capability) in response to various stimuli, including pH, light, temperature, and electric or magnetic fields. Furthermore, progress in this field of polymers has led to the development of innovative technologies such as sprayable hydrogels, nanogels, aerogels, and cryogels (Figure 3) [35,36,39,40,41].

Despite numerous advantageous properties, such as biocompatibility, biodegradability, ease of fabrication, and tunable permeability, conventional hydrogels face challenges related to low mechanical strength, which has driven the need to develop improved hydrogel formulations.

The choice of specific applications for hydrogels is determined by the structure–mechanical property relationship of polymer gels. The mechanical and swelling properties, as well as porosity of the polymer gel, are of significant importance. The mechanical properties of a polymer gel are determined by its cross-linking density and water content. These properties can be characterized through rheological analysis (which involves subjecting a sample to stress and evaluating its response to deformation) used to measure viscoelastic properties. Knowledge of swelling properties (dependent on pH, ionic strength, and temperature, and assessed using thermal analysis methods) enables the determination of the degree of cross-linking, mechanical properties, and degradation rate of polymer hydrogels. The porosity of polymer hydrogel networks plays a significant role in the applications of polymer hydrogels, as it influences the degree of swelling and the release rate of drugs. The high porosity of the hydrogel ensures high permeability to various types of active substances, making it a suitable material for controlled drug release. The porosity of polymer gels can be evaluated using various techniques, such as gas adsorption, optical microscopy, scanning electron microscopy (SEM), or transmission electron microscopy (TEM) [35,40,41,42,43].

When selecting a hydrogel and determining its potential applications, the mechanism by which its polymer network is formed, as well as the nature of the interactions involved (such as physical or chemical crosslinking), plays a significant role. Due to the transient nature of non-covalent bonds, physical hydrogels typically exhibit lower mechanical strength and stability. However, their ability to respond to stimuli such as temperature, pH, or ionic strength makes them particularly useful for applications requiring controlled substance release. The properties of physical hydrogels, including their ability to form gels under mild conditions, are advantageous for drug delivery systems and make them especially well suited for encapsulating sensitive biomolecules while preserving their functionality. Chemical hydrogels, formed through covalent cross-linking that creates stable bonds between polymer chains, exhibit superior mechanical strength and structural stability. Consequently, they are generally more durable and resistant to dissolution, making them particularly suitable for applications requiring a permanent and stable scaffold, such as tissue engineering [39,40,43].

The choice of hydrogel applications is also influenced by whether they are of natural or synthetic origin. Natural polymer hydrogels (based on polysaccharides (xanthan gum, alginate, starch, dextran, pectin, cellulose, chitosan, hyaluronic acid, carrageenan, agarose, pullulan), proteins (fibrin, collagen, gelatin, silk fibroin) or polynucleotides)) are widely used in the field of skin tissue engineering, due in part to their biocompatibility and biodegradability. Moreover, it has been shown that materials of natural origin (e.g., gelatin, pectin, starch, alginate, chitin, collagen, hyaluronates) promote cellular regeneration and tissue remodeling, and their incorporation into synthetic dressings facilitates the wound healing process [39,40,41]. Compared to natural biopolymers, synthetic polymer hydrogels (based on PEG (polyethylene glycol), PVA (polyvinyl alcohol), PAA (polyacrylic acid), PLA (polylactic acid), PVP (polyvinylpyrrolidone), PU (polyurethane), PCL (polycaprolactone), PAM (polyacrylamide), pNIPAM (poly(N-isopropylacrylamide)), PMMA (polymethyl methacrylate), and PHEMA (poly2-hydroxyethyl methacrylate)) exhibit superior mechanical properties and can be easily modified to improve their physicochemical properties. Combining natural and synthetic polymers enables the design of complex systems known as hybrid hydrogels, which integrate the advantages of both types of polymers (such as biodegradability, high strength, and the ability to control rigidity and viscosity, as well as controlled drug release). These systems often rely on multiple cross-linking strategies to improve the structural, mechanical, and functional properties of the hydrogel (Table 1) [36,37,38,39,40,41,42,43].

Hybrid hydrogels may incorporate other functional components. Various herbal extracts and plant-derived phytochemicals can be incorporated into the hydrogel matrix. Topical plant-loaded hydrogels are increasingly being investigated for their ability to support wound healing. In these formulations, the effective incorporation of plant-derived active substances into the hydrogel matrix is of key importance. Hybrid systems combining hydrogels with nanocarriers (including polymeric, metallic, and carbon nanoparticles, as well as nanoemulsions, liposomes, and other lipid nanocarriers (e.g., cubosomes, bilosomes, terpesomes, ethosomes, niosomes, glycerosomes, ufosomes, or transferosomes)) containing active plant ingredients embedded within the hydrogel structure can be classified as modern formulation strategies designed to enhance targeted release and ensure prolonged therapeutic action. Research on nanohydrogels shows that loading plant extracts into nanocarriers before their incorporation into hydrogels can improve solubility, stability, and release properties. Encapsulating plant extracts in nanocarriers protects bioactive compounds from degradation (oxidation, degradation caused by light or temperature) and enhances the stability of phytochemicals. Moreover, it may reduce local irritation by preventing burst exposure of highly concentrated phytochemicals (e.g., terpenes, essential oils) to the skin [42,43,44]. Additionally, data from the literature indicate that combining a plant extract with nanoparticles in a single formulation, such as AgNPs with antimicrobial properties or ZnO NPs, which exhibit both prohealing and antibacterial effects, enables more favorable outcomes and a multifaceted mode of action [43,44]. It is worth noting that combining a plant extract with nanocarriers does not always result in synergy; indeed, antagonistic interactions (resulting from physical, chemical, or biological effects) are sometimes observed between the extract’s active constituents and the nanocarrier. Key antagonistic phenomena include, among others, the adsorption of polyphenols onto the nanoparticle surface and their deactivation, reduced bioavailability due to excessively strong binding of active ingredients, increased system cytotoxicity, and nanoparticle aggregation leading to a loss of formulation stability [42,43,44].

The physicochemical properties of hydrogels (e.g., high porosity), which facilitate the incorporation of active compounds into the material, enable sustained release and maintain high local concentrations of active substances, making them a subject of great interest in the field of drug delivery. The release of bioactive compounds from hydrogels can occur via various mechanisms, such as swelling/deswelling, diffusion, chemical processes, and stimuli-sensitive mechanisms. One of the key parameters influencing the diffusion of active compounds within the hydrogel network is the mesh size, the value of which depends on the concentration of the polymer and cross-linking agent, as well as on external factors. When the network mesh size exceeds the size of the active molecules, the release process occurs via diffusion. In cases where the mesh size is very small and/or the active molecules are very large, they become immobilized within the network, unless they undergo degradation or the mesh size increases, for instance, in response to external stimuli. Advances in hydrogel-based drug delivery systems offer significant potential for improving the administration of bioactive compounds, as modifying hydrogel properties (e.g., water affinity, structural characteristics) enables precise control over the release and dissolution rates of therapeutic agents. These solutions could yield substantial benefits for patients with hard-to-heal wounds (e.g., diabetic foot ulcers) by enhancing the delivery of sensitive molecules crucial to the healing process [35,40,41,42,43].

### 3.2. The Use of Hydrogels in Wound Healing

Hydrogels exhibit a range of properties that make them ideally suited for use as wound dressings, representing a group of therapeutic solutions that hold great promise for improving the quality of life of many patients with wounds and their complications. They come in the form of a gel or a film and are typically transparent, allowing for easy observation of the wound surface. The three-dimensional network structure of hydrogels, resembling the natural ECM, which consists primarily of polysaccharides, proteins, and water, promotes cell adhesion, proliferation, and migration. Furthermore, they can provide a cooling effect that alleviates pain-related discomfort. Hydrogels are made from hydrophilic, swellable materials and provide a moist environment conducive to healing and represent a promising avenue for therapies supporting tissue regeneration and wound healing [35,36,37].

Healing efficacy is influenced by various physicochemical properties of hydrogels, including swelling capacity, elasticity, mechanical strength or adhesiveness. The swelling capacity of the dressing is clinically relevant for effective exudate management, ensuring increased fluid absorption. Thanks to their ability to absorb water, hydrogels can be flexible and soft, allowing them to conform to wounds located on various parts of the body. In addition to their ability to conform to shapes, hydrogels provide strong adhesion and mechanical protection, ensuring adequate coverage and safeguarding of the wound. While the benefits of using hydrogels for skin healing and regeneration are significant, certain limitations stemming from their physicochemical properties cannot be overlooked. Specifically, a high swelling capacity may lead to structural weakening of the material, distinct rheological properties may reduce dressing stability under mechanical stress, and high adhesiveness can increase the risk of trauma during dressing removal. Furthermore, increased flexibility may be associated with lower mechanical strength, while greater mechanical strength may come at the expense of reduced flexibility.

Moreover, in the context of using hydrogels for wound healing, their degradation rate is of significant importance and can be controlled to enhance their utility. Manipulating parameters such as the degree of cross-linking, incorporating bonds susceptible to hydrolysis or enzymatic degradation, and altering environmental factors, such as temperature, pressure, pH, and the concentration of active enzymes or microorganisms, allows for the control of hydrogel degradation rates. For instance, biodegradable properties of hydrogels can prevent secondary damage during dressing changes. Furthermore, the degradation rate will be a crucial factor for chronic wounds, as it influences the frequency of dressing changes. In the case of skin inflammation, for example, pH-responsive hydrogels can be designed to degrade more rapidly in the acidic environment found in inflamed tissue [35,36,37,40].

Designed for topical application, hydrogels are best suited for wounds with moderate to low exudate, as they isolate bacteria, tissue debris, and odor-causing molecules within their structure by absorbing and retaining contaminated exudate, thereby helping to maintain a cleaner wound environment. They are also suitable for use on dry wounds, as they create a moist environment that promotes autolytic debridement of necrotic tissue. Hydrogels are used for the treatment of acute wounds (e.g., burns, surgical wounds, and traumatic injuries) as well as hard-to-heal wounds, including pressure ulcers, diabetic foot ulcers, venous leg ulcers, infected wounds, and chronic ulcerations (Table 2) [35,36,37,38,39].

Due to their advantages, various polymers can be used in hydrogel dressings. These include, among others [36,37,39,41,43], the following:

Alginate: Forms hydrogels through ionic cross-linking with calcium ions; exhibits structural similarity to the ECM; biocompatible, non-toxic, microbiologically biodegradable, and cost-effective; features high water solubility, rapid gelation kinetics and excellent plasticity; can activate macrophages and stimulate the production of IL-6 and TNF-α; provides a moist environment conducive to wound healing and accelerates the healing of chronic wounds; allows for the painless and safe removal of dressings.

Gelatin: A polymer with properties beneficial for tissue development (bioactivity, biocompatibility, low immunogenicity); creates a favorable environment for cell proliferation; the presence of functional groups allows for the modification of the material using cross-linking agents or therapeutic substances; is inexpensive and highly degradable; material approved by the FDA for medical applications.

Hyaluronic acid: A glycosaminoglycan naturally occurring in skin tissue that promotes hydration, cell adhesion, and cellular regeneration; enzymatically biodegradable, biocompatible, non-toxic, non-immunogenic; offers a suitable solution at each of the four stages of wound healing due to properties such as reducing inflammation, enhancing angiogenesis, and stimulating endothelial cell growth; effectively absorbs exudate; is characterized by high viscosity and mechanical stability.

Chitosan: A cationic polysaccharide; due to its positive charge, it is used to coat nanoparticles to enhance their stability; enzymatically and microbiologically biodegradable, non-toxic; characterized by structural similarity to extracellular matrix GAGs, biological inertness, low antigenicity, a tunable degradation rate; maintains a moist wound environment, protects against infection, stimulates leukocyte activity in clearing exudate, exhibits antibacterial and hemostatic properties, improves cell adhesion, and reduces scar formation.

Cellulose: The most abundant renewable polymer on Earth; its derivatives are valued for their biocompatibility, biodegradability, low toxicity, and cost-effectiveness; they exhibit natural wound-healing properties; they are used to improve viscosity, swelling capacity, and application characteristics, including spreadability.

Polyethylene glycol: A non-ionic polymer; non-toxic and biocompatible; can act as a cross-linking agent due to its rigidity, water solubility, and low immunogenicity; characterized by high hydrophilicity, flexibility, and a high degree of drug encapsulation; can be easily modified with other polymers.

Polyvinyl alcohol: Biocompatible, non-toxic, conditionally biodegradable; exhibits swelling capacity and bioadhesive properties; characterized by excellent water and oxygen permeability and the ability to maintain high moisture levels; protects wounds against external factors and mechanical trauma, thereby reducing the risk of secondary injury.

Polyvinylpyrrolidone: A crystalline polymer soluble in water and polar solutions; biocompatible and non-toxic; capable of absorbing water up to one hundred times its own mass; allows for the selective permeation of oxygen while blocking the entry of bacteria and other contaminants; protects the wound from further damage or infection; aids in the wound-cleansing process by effectively absorbing exudate and necrotic tissue; improves the bioavailability of poorly water-soluble active compounds.

Despite the widespread use of hydrogels in topical therapy, further research is needed, including hydrogel optimization and clinical trials, to evaluate the long-term safety of these materials and their efficacy in wound healing [39,41].

## 4. Application of Asteraceae and Lamiaceae Families Plant-Extract-Incorporated Hydrogels in Wound Healing and Skin Regeneration—Bibliometric Analysis of Trends

### 4.1. Materials and Methods

Database searches were conducted in PubMed/Medline, Web of Science Core Collection, and Scopus to identify articles published between 2022 and 2026 (last searched on 22 May 2026). Search strategies were conducted using the following free-text keywords: “Asteraceae”, “Lamiaceae”, “plant extract”, “skin wound healing”, and “hydrogel”, and terms related to the research topic were combined using logical operators (AND/OR) to ensure a comprehensive search of the databases. The results were reported in accordance with the PRISMA 2020 Guidelines (Preferred Reporting Items for Systematic Reviews and Meta-Analyses) [45]. This systematic review was not prospectively registered and no formal review protocol was prepared.

Study eligibility criteria were defined before the start of the study, in accordance with pre-established inclusion and exclusion criteria, to ensure greater precision and accuracy. Inclusion criteria: (i) full-text original experimental studies (in vitro and in vivo studies, as well as clinical studies) published in academic peer-reviewed journals; (ii) studies concerning the efficacy of hydrogels containing plant extracts from the Asteraceae and Lamiaceae families in skin wound healing and regeneration; (iii) articles published in English.

Exclusion criteria: (i) review articles, conference abstracts, meetings, letters, notes, or editorials were excluded; (ii) research on hydrogels based on bioactive compounds isolated from plants (e.g., polysaccharides, polyphenols), or plant-based products (e.g., essential oils, hydrolate, oils, juice, honey, fermented products, mucilage); (iii) studies investigating hydrogel formulations containing polyherbal extracts because the individual contribution of an extract from a specific plant belonging to the Lamiaceae or Asteraceae families to wound healing and skin regeneration outcomes could not be clearly determined; (iv) research unrelated to hydrogel-based formulations; (v) studies focused on application unrelated to wound healing or skin regeneration.

For data synthesis, studies were grouped according to the plant family (Asteraceae or Lamiaceae), plant part and extraction method, extract type, origin and type of polymer used for preparing hydrogel, experimental model (in vitro, in vivo, or clinical), and reported wound-healing and skin-regeneration outcomes.

Study selection was performed by a single reviewer according to predefined eligibility criteria. Potential uncertainties regarding study eligibility were resolved through repeated assessment of the full-text articles. No automation tools were used during the screening and study selection process. The selection process is illustrated in the PRISMA flow chart (Supplementary Figure S1). After eliminating duplicate studies, the titles and abstracts of all relevant articles were initially screened to determine whether they met the inclusion criteria. Data were extracted from all eligible studies and the information collected included authors and year of publication, country, plant family and species, solvent used, extract type, extraction method, extract chemical composition, hydrogel formulation, biological activity, experimental model (in vitro, in vivo, or clinical), wound type, evaluated outcomes, and key conclusions. The primary outcomes of interest were wound-healing and skin-regeneration effects associated with hydrogels containing plant extracts. Data were collected on outcomes including wound closure rate, stimulation of skin cell viability and proliferation, re-epithelialization, granulation tissue formation, collagen deposition, angiogenesis, anti-inflammatory, antioxidant, and antimicrobial activity, and cytocompatibility. Additional outcomes included physicochemical properties of the hydrogels, such as swelling capacity, mechanical strength, degradation behavior, porosity, and release profiles of bioactive compounds.

The methodological quality of the included studies was assessed using predefined criteria appropriate for each study type. The assessment considered the clarity of hydrogel characterization, description of the plant extract, experimental design, presence of appropriate controls, and outcome reporting. The findings were subjected to a descriptive synthesis and presented according to the reported wound healing, skin regeneration, and physicochemical and biological outcomes. Due to the methodological heterogeneity and the variation in results among the included studies, subgroup analyses, meta-regression, and sensitivity analyses were not performed because a quantitative meta-analysis was not conducted. In this narrative synthesis, sources of heterogeneity were explored qualitatively. The characteristics and findings of the included studies are summarized in tables and figures. Data are presented according to plant family and species, extract characteristics, type of hydrogel formulation, experimental model, biological activities, and reported wound-healing outcomes. A formal assessment of reporting bias was not conducted because the included studies were heterogeneous and no meta-analysis was performed. The possibility of publication bias was considered during data interpretation; however, a formal assessment was not conducted due to the diversity of study designs, outcomes, and reporting methods. Due to the predominance of in vitro and animal studies, a formal assessment of certainty of evidence was not performed. The overall strength of the evidence was considered qualitatively based on study design, methodological quality, consistency of findings, and the characteristics of the experimental models used.

### 4.2. Results and Discussion

The primary search provided 721 published articles. After removing 81 duplicates, 640 records remained for title and abstract screening. The first screening phase led to the exclusion of 447 records. The remaining 193 articles were rigorously assessed against eligibility criteria. A total of 169 full-text articles were excluded, and the most common reasons were (1) irrelevant focus or study objective, n = 68; (2) insufficient data for efficacy assessment, n = 62; and (3) insufficient material/matrix characterization (n = 39). Ultimately, 24 studies satisfied all inclusion criteria and were included in a final synthesis. Details of the selection process are shown in the PRISMA flow diagram (Supplementary Figure S1).

**Figure 4:**
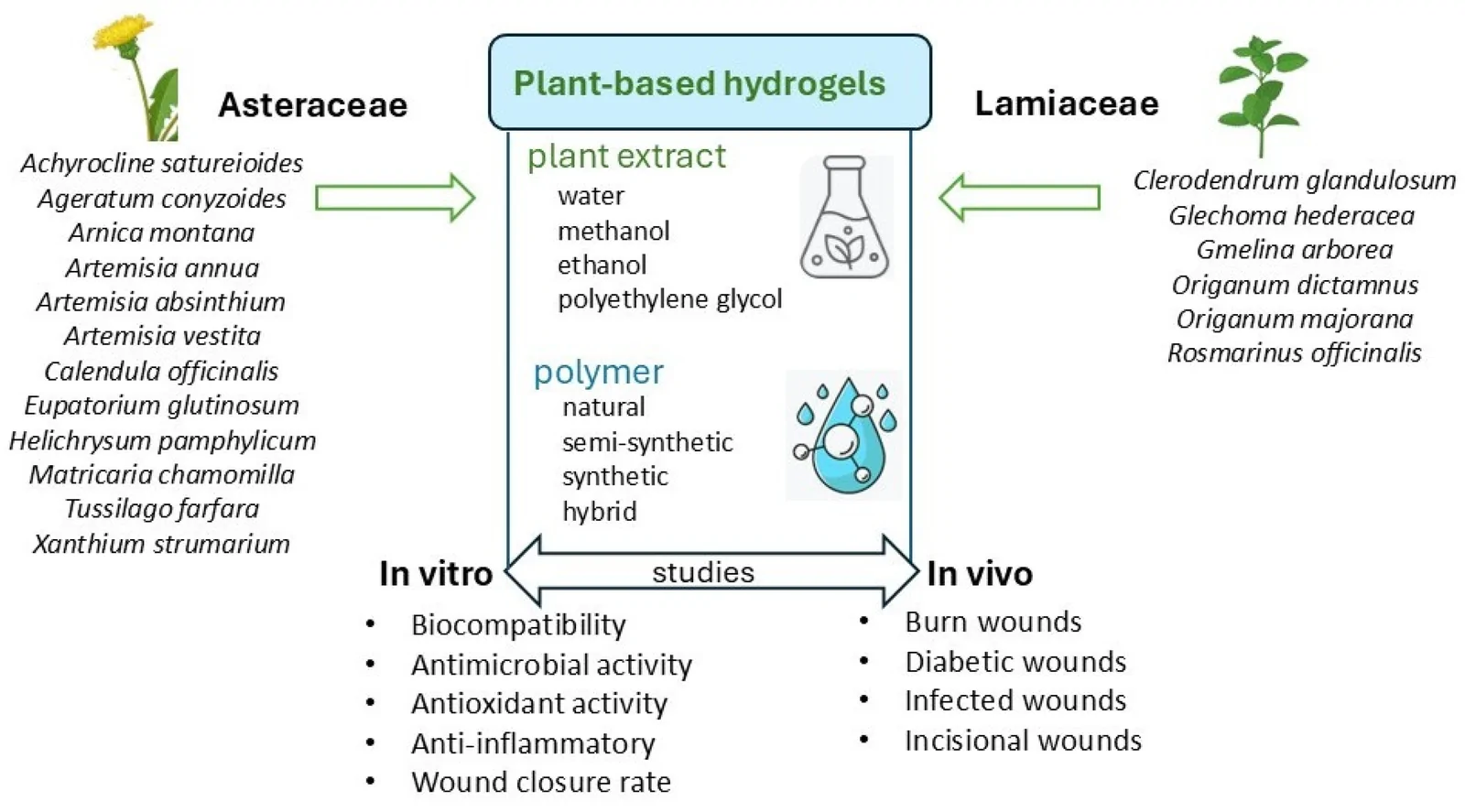
The latest findings on hydrogels containing extracts from Asteraceae and Lamiaceae species tested for wound healing and skin regeneration.

**Figure 5:**
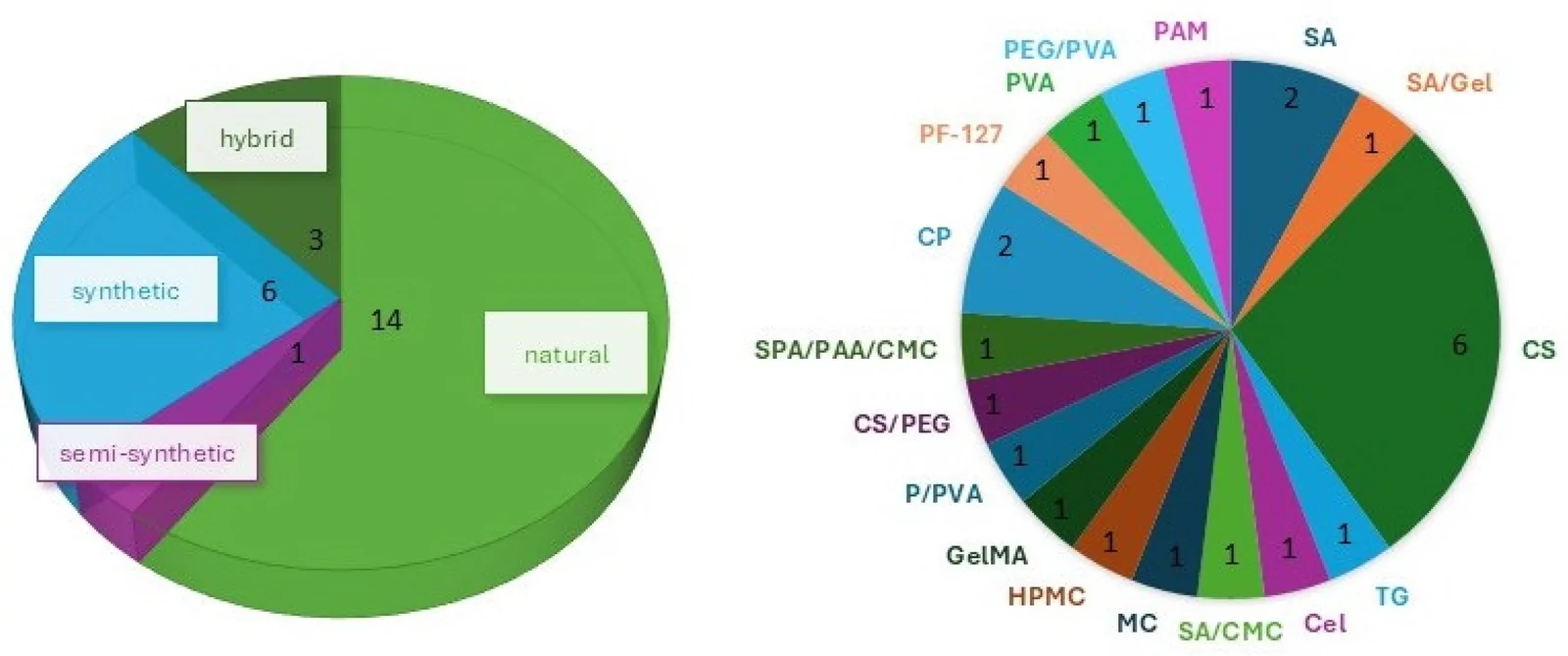
Research trends in different hydrogel formulations among the included studies. Cel, cellulose; CMC, carboxymethylcellulose; CP, carbopol; CS, chitosan; Gel, gelatin; GelMA, gelatin methacryloyl; HPMC, hydroxypropylmethylcellulose; MC, methylcellulose; P, pullulan; PAA, polyacrylic acid; PAM, polyacrylamide; PEG, polyethylene glycol; PVA, polyvinyl alcohol; SA, sodium alginate; SPA, sodium polyacrylate; TG, tragacanth gum, PF-127, Pluronic F-127.

The detailed extracted data are summarized in Table 3, Table 4 and Table 5 and presented in Figure 4 and Figure 5. Overall, the included studies were of moderate to high methodological quality. Most studies presented detailed information on the hydrogel preparation and evaluation of biological outcomes. Three publications failed to provide sufficient information regarding the extraction method. Some studies lacked a comprehensive phytochemical characterization of the extracts and failed to specify extraction yields or the quantitative recovery of key bioactive compounds. Furthermore, some articles did not include physicochemical analyses of the hydrogel formulations. Due to substantial heterogeneity regarding plant species, tested extracts, hydrogel formulations, experimental models, outcome measures, and reporting methods, a quantitative meta-analysis was not performed. Therefore, findings were synthesized narratively, and sensitivity analyses were not applicable. For these reasons, a formal assessment of reporting bias was not conducted. Across the included in vitro and in vivo studies, hydrogels containing plant extracts generally demonstrated beneficial effects on wound healing and skin regeneration, including accelerated wound closure, enhanced cell proliferation, and antimicrobial, antioxidant, or anti-inflammatory activity. Confidence in the available evidence is limited by the predominance of preclinical studies (in vitro and animal studies), variability in study design, and inconsistencies in outcome reporting.

#### 4.2.1. Characteristics of Plant Material from the Asteraceae and Lamiaceae Families in the Analyzed Studies

The present review revealed that research on plants from the Asteraceae and Lamiaceae families used to prepare plant extracts was conducted by scientists from various regions of the world, including Poland, Romania, Türkiye, Ecuador, Singapore, China, India, Iran, Hungary, Korea, Brazil and México. The most commonly used plant, among species belonging to the Asteraceae (*Achyrocline satureioides*, *Ageratum conyzoides*, *Arnica montana*, *Artemisia annua*, *Artemisia absinthium*, *Artemisia vestita*, *Calendula officinalis*, *Eupatorium glutinosum*, *Helichrysum pamphylicum*, *Matricaria chamomilla*, *Tussilago farfara*, *Xanthium strumarium*) and Lamiaceae (*Clerodendrum glandulosum*, *Glechoma hederacea*, *Gmelina arborea*, *Origanum dictamnus*, *Origanum majorana*, *Rosmarinus officinalis*) families, incorporated in the hydrogel tested for wound healing was *Calendula officinalis*. The frequency of selecting this plant for research may be related to the fact that calendula has a long tradition of use in folk medicine, has been used in Europe to treat wounds since the 13th century, and its use as a medicinal agent is supported by various in vivo and in vitro studies [15].

The studies included in this review employed various solvents, such as water, ethanol, methanol, and polyethylene glycol, to obtain extracts from diverse plant parts, including aerial parts, leaves, inflorescences, flowers, and petals. Moreover, various extraction methods were used to obtain plant extracts, which were incorporated into the tested hydrogels (Table 3).

**Table 3:** Characterization of plant material from Asteraceae and Lamiaceae found within the scope of this review.

Lp.	Species	Plant Part	Extract Type	Extraction Method	Ref.
Asteraceae Family					
1.	*Achyrocline satureioides* (Lam.) DC	inflorescences	WEE	maceration, 8 days	[26]
2.	*Ageratum conyzoides* L.	leaf	WEE	UAE	[46]
3.	*Arnica montana* L.	aerial parts	EE	ns	[27]
4.	*Artemisia absinthium* L.	leaf	WEE	MSE, 12 h, room temperature	[34]
5.	*Artemisia annua* L.	aerial parts	WEE	commercial dried extract, DER 1:30	[47]
6.	*Artemisia vestita* Wall. ex Besser	leaf	WE	Soxhlet extraction	[48]
7.	*Calendula officinalis* L.	aerial parts	WEE	maceration, 72 h and percolation, 5 days	[49]
8.	*Calendula officinalis* L.	flower	WE	HAE, 60 °C for 30 min	[50]
9.	*Calendula officinalis* L.	flower	WEE	maceration, 48 h	[33]
10.	*Calendula officinalis* L.	flower	WEE	commercial extract	[51]
11.	*Calendula officinalis* L.	flower	WEE	HAE, 95 °C, 3 × 30 min	[52]
12.	*Calendula officinalis* L.	flower petals	PEG-WE	maceration, room temperature, 14 days, intermittent stirring	[30]
13.	*Calendula officinalis* L.	ns	EE	maceration, 24 h followed by UAE, 15 min	[53]
14.	*Eupatorium glutinosum* Lam.	leaf	ME, EE,	maceration, 15 days, room temperature	[54]
15.	*Helichrysum pamphylicum* P.H. Davis & Kupicha	aerial parts	ME	UAE, 25 °C, 3 × 2 h	[55]
16.	*Matricaria chamomilla* L.	flower	WEE	SAE, 25 °C, 48 h	[56]
17.	*Tussilago farfara* L.	leaf	WEE	maceration, 72 h, 25 °C, constant stirring at 150 rpm	[57]
18.	*Xanthium strumarium* L.	ns	WE	UAE, 65 °C for 1 h	[58]
	Lamiaceae Family				
19.	*Clerodendrum glandulosum* Lindl.	leaf	WE	HAE, 60–70 °C	[59]
20.	*Glechoma hederacea* L.	aerial parts	ME	ns	[60]
21.	*Gmelina arborea* Roxb.	leaf	WE	HAE, 50 °C, 30 min	[61]
22.	*Origanum dictamnus* L.	aerial parts	WE	infusion	[32]
23.	*Origanum majorana* L.	leaf	WE	HAE, 50 °C, 48 h	[62]
24.	*Rosmarinus officinalis* L.	aerial parts	WEE	maceration, room temperature, 10 days	[63]

WE, water extract; ME, methanol extract; EE, ethanol extract; WEE, water–ethanol extract; PEG-WE, polyethylene glycol-water extract; DER, drug-to-extract ratio; HAE, heat-assisted aqueous extraction; MSE, magnetic stirrer extraction; SAE, shaker-assisted extraction; UAE, ultrasound-assisted extraction; ns, not specified.

Despite the use of various extraction methods, including maceration, Soxhlet extraction, magnetic stirring extraction, shaker-assisted extraction, and ultrasound-assisted extraction, comparing specific techniques remains challenging, as many studies fail to specify process yields or the quantitative recovery of key bioactive compounds. In the articles under discussion, phytochemical characterization was performed only on selected plant extracts, using a variety of analytical techniques. The yield of the methanolic extract from *Helichrysum pamphylicum* was 4.0% [55]. The UFLC–PDA (ultra-fast liquid chromatography with photodiode array detector) technique was used to determine such flavonoid aglicones as quercetin (296.5 μg/mL), luteolin (153.70 μg/mL), and 3-*O*-methylquercetin (602.0 μg/mL) in the *Achyrocline satureioides* extract [26]. The presence of such active compounds as chlorogenic acid, narcissin (isorhamnetin 3-*O*-rutinoside), and typhaneoside (isorhamnetin 3-*O*-rhamnosylorutinoside) in *Calendula officinalis* extract was determined by using UHPLC–MS/MS (ultra-high-performance liquid chromatography–mass spectrometry and UHPLC–DAD (ultra-high-performance liquid chromatography with diode array detector) methods. The plant material met the requirements of the Pharmacopoeia regarding total flavonoid content, which was 0.95% in the dried flowers [52]. In other studies, TFC in dried calendula flowers was 0.76% (expressed as hyperoside), and in the glycolic extract, it was 1.66% (expressed as quercetin) [30]. In the aqueous–ethanolic extract of *Rosmarinus officinalis*, the total polyphenol content was 75.20 mg/g, expressed as gallic acid equivalents [63]. In *Artemisia absinthium* extract, 15 phenolic compounds were identified using LC–MS/MS (liquid chromatography–tandem mass spectrometry), including thymoquinone (762.06 μg/L), resveratrol (717.06 μg/L), oleuropein (79.82 μg/L), myricetin (35.04 μg/L), quercetin (268.79 μg/L), butein (196.97 μg/L), luteolin (57.07 μg/L) and curcumin (15.09 μg/L), as well as phenolic acids such as acetohydroxamic (315.55 μg/L), syringic (500.69 μg/L), fumaric (3593.71 μg/L), gallic (421.76 μg/L), caffeic (120.71 μg/L), 4-hydroxycinnamic (37.31 μg/L), and ellagic acid (130.1 μg/L) [34]. In another study, LC–MS/MS analysis of *Glechoma hederacea* extract revealed the identification of such metabolites as rosmarinic acid, ursolic acid, 2*α*-hydroxyursolic acid, 2*β*-hydroxyursolic acid, and luteolin 7-*O-β*-D-glucopyranoside [60]. The LC–MS method was also used to identify compounds such as flavonoids, polyphenols (caffeic, neochlorogenic, 4,5-dicaffeoylquinic, 3-methoxycinnamic, 2-oxo-2H-chromene-3-carboxylic acid and scoparone), amino acids and peptides (L-tryptophan, leucylproline), as well as other metabolites as trigonelline, phytic acid, *β*-lapachone, 4-oxo-5-phenylpentanoic acid, sucrose, itaconic acid, xanthosine, pantothenic acid, azelaic acid, and hexanoic acid present in the extract of *Xanthium strumarium* [58]. Selected bioactive constituents of the *Artemisia annua* extract were quantified using LC–ESI–MS (liquid chromatography–electrospray ionization mass spectrometry), including casticin (108.36 ng/mg) and artemetin (15.83 ng/mg), as well as sesquiterpenoids such as artemisinin (traces), arteannuin B (2.30 ng/mg), deoxyartemisinin (24.92 ng/mg), and dihydroartemisinic acid (11.03 ng/mg) [47]. LC–MS analysis identified such flavonoids as luteolin, kaempferol, quercetin, isoquercitin, rutin, and astragalin, while GC–MS (gas chromatography–mass spectrometry) analysis confirmed the presence of secondary metabolites as terpenes, sterols, aliphatic alcohols, esters, fatty acids, and aldehydes in *Gmelina arborea* extract [61]. The analysis of *Artemisia vestita* extract using GC–MS revealed 22 compounds, including grandisol, camphor, 1,8-cineol, limonene, thujone, β-caryophyllene, isofraxidin, naringenin, myrcene, camphene, apigenin, santolina triene, α-pinene, germacrene-D, yomogin, artemisia alcohol, cirsilineol, borneol, quercetin, copaene, artemisia ketone, and amyrin [48]. The identification of bioactive constituents, such as monoterpenoids, sesquiterpenoids, phenol, alkaloid, diterpenoid, amino acid, and two biomacromolecules in the *Origanum majorana* extract was performed using GC–MS [62]. The extraction yield of *Eupatorium glutinosum* was 8.97% using methanol as the solvent, while it was 6.37% using ethanol. Quaternary alkaloids, saponins, terpenoids, and phenolic compounds were detected in extracts using FTIR (Fourier-transform infrared spectroscopy) and TLC (thin-layer chromatography), as well as through UV–Vis profile analysis [54].

#### 4.2.2. Characterization of Hydrogels Containing Extracts from Plants of the Asteraceae and Lamiaceae Families

The review showed that diverse materials were used to prepare hydrogels, including natural polymers (e.g., chitosan, tragacanth gum, sodium alginate, gelatin, cellulose and its derivatives) in 14 studies, semi-synthetic polymers (methacryloyl gelatin) in 1 study, synthetic polymers (e.g., polyvinyl alcohol, polyethylene glycol, carbomer, polyacrylamide, Pluronic F-127) in 6 studies, or hybrid materials (e.g., chitosan/polyethylene glycol, pullulan/polyvinyl alcohol, sodium polyacrylate/polyacrylic acid/carboxymethylcellulose) in 3 studies (Figure 5). Moreover, various formulation strategies, including simple as well as more advanced and modern formulation strategies, including physical incorporation of the extract into the hydrogel, encapsulation of the extract in carriers before introduction into the hydrogel, methods based on nanostructures, and 3D printing of hydrogels with the extract, have been used to prepare extract-based hydrogels.

In total, 2 of the 24 analyzed publications tested hydrogels prepared using sodium alginate. Papp et al. [47] evaluated an alginate hydrogel with *Artemisia annua* extract prepared using a water–ethanol solvent, while Possa et al. [30] tested an alginate hydrogel with *Calendula officinalis* flower petals macerated in a solution of polyethylene glycol and water. Moshfeghi et al. [56] prepared a sodium alginate/gelatin hydrogel comprising *Matricaria chamomilla* flowers with a 70% water–ethanol extract. In the subsequent six studies, hydrogels prepared using another natural polymer, chitosan, were employed. Chanaj-Kaczmarek et al. [52] used two types of chitosan with different viscosity in combination with an ethanol–water extract from *Calendula officinalis* flowers to obtain a hydrogel delivery system. Ismailovi et al. [55] conducted studies on chitosan gel formulations prepared from chitosan polymer at different rates and molecular weights, and mixed with a methanol extract obtained from the aerial parts of *Helichrysum pamphylicum*. Aydin et al. [34] synthesized a hydrogel using low-molecular-weight chitosan and 70% water–ethanol extract from *Artemisia absinthium* leaves. Nakamura-García et al. [34] prepared the matrix for the study by mixing chitosan hydrogel with the plant extracts obtained by maceration of *Calendula officinalis* flowers in 95% ethanol aqueous solution. Naseriyeh et al. [53] prepared a chitosan-based hydrogel with nanoliposomes containing ethanolic extract of *Calendula officinalis* powder. Rodríguez-Acosta et al. [50] developed chitosan-based hydrogels loaded with silver nanoparticles and aqueous extract of *Calendula officinalis* flower petals. In the next study, another natural polymer was used to prepare a hydrogel based on tragacanth gum and an aqueous extract of *Artemisia vestita* leaves obtained by Soxhlet extraction [48]. Among the semi-synthetic polymers, one of the studies discussed in this review used cellulose to prepare a hydrogel loaded with the ethanolic and methanolic extracts obtained by maceration of *Eupatorium glutinosum* leaves [54]. In contrast, the second study used methyl cellulose to prepare a hydrogel with *Glechoma hederacea* herb methanolic extract [60]. Another study focused on the preparation of a hydrogel based on sodium alginate, carboxymethylcellulose, and 70% water–ethanol extract of *Tussilago farfara* leaves incorporated into a hydrogel matrix [57]. The next test was conducted on *Xanthium strumarium* water extract incorporated into gelatin methacryloyl (semi-synthetic modified natural polymer) hydrogel [58]. Five research teams tested hydrogels prepared using synthetic polymers. In the study by Kantesaria et al. [46], polyvinyl alcohol (PVA) was used as the hydrogel matrix, and 70% water–ethanolic extract of *Ageratum conyzoides* leaves, obtained via ultrasound-assisted extraction, was employed as the bioactive component. In Pelin et al.’s [51] study, hydrogels composed of pullulan (a natural polysaccharide) and polyvinyl alcohol (P/PVA) were prepared and subsequently loaded with the hydroalcoholic extract of *Calendula officinalis* using a simple post-loading immersion method. In Mohammadi et al.’s [62] article, an aqueous extract was prepared by maceration from *Origanum majorana* leaves and encapsulated into the polymer (polyethylene glycol (PEG)/polyvinyl alcohol (PVA) matrix. Gavan et al. [63] prepared a carbomer-based hydrogel loaded with *Rosmarinus officinalis* aerial parts extract obtained by maceration in 70% water–ethanol solvent. Balestrin et al. [26] used a carbopol-based hydrogel containing 80% hydroethanolic extract from inflorescences of *Achyrocline satureioides* obtained by maceration in ethanol incorporated in a nanoemulsion. In a subsequent study, a polyacrylamide hydrogel synthesized via free-radical polymerization was used, incorporating a calendula 70% water–ethanol extract obtained by the percolation method [49]. Chandrasekharan et al. [61] used an aqueous extract of *Gmelina arborea* leaves to prepare a PF-127 hydrogel (known as Pluronic F-127, a synthetic, non-ionic triblock copolymer) with an extract of silver nanoparticles. Majie et al. [59] formulated a chitosan- and polyethylene glycol (PEG)-based hydrogel loaded with silver nanoparticles synthesized from the aqueous extract of *Clerodendrum glandulosum* leaves. Finally, it is worth mentioning two studies in which hydrogel patches were evaluated. Paloukopoulou et al. [32] used hydroxypropylmethylcellulose (HPMC) to develop 3D-printed hydrogel patches containing *Origanum dictamnus* infusion, while Lee et al. [27] tested a hydrogel patch based on a hybrid polymer network composed of polyacrylic acid, sodium polyacrylate, and cellulose gum (carboxymethylcellulose), incorporating *Arnica montana* extract as the bioactive component.

In addition to assessing the properties related to the action and influence of extract-based hydrogels on the wound-healing process in in vitro and in vivo studies (Table 4 and Table 5), the authors also tested the physicochemical properties (e.g., gelation time, water-retaining capacity, moisture content, pH, swelling ratio), rheological and textural analysis, and morphological properties (e.g., thickness, surface, and cross-sectional images, the pore size, porosity, and distribution of pores) using light microscopy (SEM and TEM), chemical structure analysis, and confirming successful incorporation using FTIR (Fourier-transform infrared spectroscopy) of matrices used in the study, as well as the release of plant materials from the matrix network. Conducting such studies and evaluating the physical, chemical, and mechanical parameters of hydrogels, whether natural, synthetic, or hybrid, is important for their potential use in treating various types of wounds. For instance, studies on a polyvinyl-alcohol-based hydrogel containing *A. conyzoides* extract demonstrated that the phytoextract increases pore size (FESEM analysis) and modifies the hydrogel’s thermal properties (TGA and DSC analysis) while preserving its elasticity, a key characteristic for wound dressings. The incorporation of phytochemicals reduced the hydrogel’s water-retention capacity. Furthermore, the material retained its structural integrity upon full swelling, assuming a soft, gelatinous form without disintegrating. These properties make hydrogels highly advantageous formulations for the topical treatment of wounds, as they can protect the surrounding skin from maceration while maintaining a moist wound environment, which promotes healing and prevents scarring [46]. Studies on a chitosan hydrogel containing calendula-loaded liposomes have shown that the addition of liposomes increases absorption capacity and improves water vapor permeability within the granulation tissue forming on the wound surface, while the processes of release and dermal absorption occur in a slow, controlled manner, demonstrating that this dressing could be a suitable solution for promoting rapid wound healing [53]. Subsequent studies evaluating swelling properties confirmed that the SA/CMC/ZnONPs/*T. farfara* hydrogel enables exudate absorption, thereby creating a favorable environment for cell migration and proliferation as well as the delivery of active substances. It was found that the addition of ZnO nanoparticles and *T. farfara extract* did not significantly affect the hydrogel’s physical properties, including its degradation profile and water-absorption capacity. Given these findings, the alginate- and cellulose-based dressing, containing a nanocarrier and plant extract, can be recommended for the treatment of burn wounds [57]. Other studies have confirmed that a hydrogel based on chitosan and polyethylene glycol, loaded with active ingredients such as *C. glandulosum*-AgNPs, exhibited excellent functional properties (particle size, zeta potential, high viscosity ensuring good mucoadhesion, as well as slow and complete degradation providing sufficient time for sustained release), making it a suitable material for the treatment of chronic wounds [59].

#### 4.2.3. In Vitro Studies Evaluating Hydrogels Incorporating Asteraceae and Lamiaceae Plant Extracts

Table 4 summarizes the results of in vitro studies on hydrogels containing different plant extracts from the Asteraceae and Lamiaceae families. The research results show that the effectiveness of hydrogels based on plant extracts is related to their antimicrobial and antibiofilm activity, antioxidant and anti-inflammatory effects, and stimulation of skin cell viability and proliferation.

**Table 4:** In vitro studies evaluating plant-extract-loaded hydrogels.

Preparation Form	Cell Model	Method	Key Results	Ref.
*Ageratum conyzoides* extract-loaded PVA hydrogel	*-S. aureus, E. coli, A. niger;* -L929 cell line	-MTT assay -agar disk diffusion method	-biocompatibility with fibroblast cells -antibacterial activity	[46]
*Artemisia annua* extract-loaded SA hydrogels	-cell-free assay -HaCaT cell line	-MTT assay -scratch assay -DPPH, FRAP, CUPRAC	-biocompatibility with keratinocytes; ↑ keratinocyte migration, ↑ wound closure -antioxidant activity	[47]
*Artemisia vestita* extract-loaded TG hydrogel	*-S. aureus, B. subtilis, E. coli, S. pyogenes, C. albicans, A. niger, A. flavus* -HaCat cel line	-agar well diffusion method, MIC, MBC/MFC; -scratch assay	-antimicrobial activity -~95% wound closure	[48]
*Artemisia absinthium* extract-loaded CS hydrogel	*S. aureus, P. aeruginosa*	disk diffusion method	no antibacterial action	[34]
*Calendula officinalis* extract-loaded P/PVA hydrogels	-*S. aureus, E. coli, P. aeruginosa, C. albicans* -HDF cell line -cell-free assay	-disc diffusion method, TKA -MTT assay -DPPH assay	-antimicrobial properties -non-cytotoxic against fibroblast cells -antioxidant activity	[51]
Nanoliposomes containing *Calendula officinalis* extract-loaded CS hydrogel	L929 cell line	MTT assay	↑ proliferation, viability, biocompatibility with fibroblasts	[53]
*Calendula officinalis* extract-loaded CS hydrogel	*S. aureus, S. epidermidis, E. faecalis, E. faecium,* *S. pyogenes, E. coli, P. aeruginosa, P. acnes, C. albicans*	agar diffusion method, MBC	bactericidal effect against *S. aureus, P. acnes, E. coli*	[52]
*Calendula officinalis* extract -loaded SA hydrogel	3T3 cell line	MTT and SRB assays	↑ fibroblast cell viability	[30]
*Clerodendrum glandulosum* extract-AgNP-loaded CS-PEG hydrogel	*-E. coli, S. aureus* -cell-free assay	-disc diffusion method -DPPH, ABTS	-antimicrobial properties -antioxidant activity	[59]
*Eupatorium glutinosum* extract-loaded Cel hydrogel	*E. coli*	agar diffusion method	antibacterial effect	[54]
*Gmelina arborea* extract-AgNP PF-127 hydrogel	*-B. cereus, E. coli*, *P. aeruginosa*, *S. aureus,* -HDF cell line	-disc diffusion method, MIC, MBC assay, CV assay; -scratch assay	-antibacterial and antibiofilm activity;- -↑ wound closure rate	[61]
*Helichrysum pamphylicum* extract-loaded CS- hydrogel	cell-free assay	DPPH, ABTS, metal chelating	antioxidant activity	[55]
*Matricaria chamomilla* extract-loaded SA/Gel hydrogel	-L929 cell line *-S. aureus* (Gram-positive) and *E. coli*	-MTT assay -disc diffusion method	-biocompatibility with fibroblast cells -no inhibitory effect against bacteria	[56]
HPMC hydrogel patches with *Origanum dictamnus* extract	HaCaT cell line	scratch assay, qPCR assay	~70% wound closure; anti-inflammatory (↓ IL-6 and TNF-α, ↑ IL-4 level)	[32]
*Origanum majorana* extract-loaded PVA–PEG matrix	*-S. aureus* and *E. coli* *-* HSF cell line	-disc diffusion method -MTT assay, microscopic observations	-antibacterial activity -↑ fibroblast adhesion, proliferation, and distribution;	[62]
*Rosmarinus officinalis* extract-loaded CP hydrogel	*E. coli, S. aureus, P. aeruginosa, C. albicans*	disc diffusion method	antimicrobial activity (*S. aureus, P. aeruginosa, C. albicans*)	[63]
*Tussilago farfara* extract-loaded SA/CMC hydrogel	-3T3 cell line -*S. aureus, P. aeruginosa*	-MTT assay -TKA	-biocompatibility with fibroblast cells -antibacterial activity	[57]

↑ Improved, enhanced, increased; ↓ decreased, reduced; CS, chitosan; PVA, polyvinyl alcohol; P, pullulan; AgNP, silver nanoparticle; PEG, polyethylene glycol; SA, sodium alginate; Gel, gelatin; TG, tragacanth gum; Cel, cellulose; PF-127, Pluronic F-127; HPMC, hydroxypropylmethylcellulose; CP, carbopol; CMC, carboxymethylcellulose; MIC, minimum inhibitory concentration; MBC, minimum bactericidal concentration; MFC, minimum fungicidal concentration; CV, crystal violet; TKA, time kill assay; MTT, 3-(4,5-dimethylthiazol-2-yl)-2,5-diphenyltetrazolium bromide); SRB, sulforhodamine B.

In the evaluation of topical preparations, cytotoxicity assessment is a key stage in assessing safety and efficacy. Studies on an alginate hydrogel containing 10% *Calendula officinalis* extract confirmed that the material exhibited no cytotoxic activity (MTT and SRB assays) against fibroblast cells, but, instead, promoted cell proliferation, indicating its potential to support cellular processes essential for wound healing [30]. Other in vitro studies on fibroblast cells (MTT assay) demonstrated no cytotoxicity of SA/gel hydrogel with *M. chamomilla* extract [56], SA/CMC hydrogel with *Tussilago farfara* extract [57], chitosan-based hydrogel with nanoliposomes containing *C. officinalis* extract [53], or of P/PVA hydrogels enriched with *C. officinalis* extract, and confirmed the cytocompatibility of this formulation [51]. In other studies, the authors, summarizing the results of the MTT cytotoxicity assay, concluded that the cytotoxicity of citric-acid-crosslinked PVA hydrogel could be reduced by incorporating *Ageratum conyzoides* extract [46]. The study of fibroblast cell viability using the MTT assay indicated that the *Origanum majorana* extract-loaded PVA–PEG matrix is compatible with cells and promotes cell growth [62]. In the study of keratinocyte cell viability using the MTT test, it was shown that the *Artemisia annua*-loaded alginate hydrogels ensured high cell viability, indicating enhanced metabolic activity and biocompatibility. Furthermore, extract-based formulations, especially those additionally containing D-panthenol and hyaluronic acid, promoted cell migration and proliferation and accelerated wound healing, facilitating the closure of the scratch area [47]. Wound-healing potential was also confirmed by a cell scratch assay in human dermal fibroblast cell lines for *Gmelina arborea* extract-AgNP PF-127 hydrogel [61].

In the context of wound healing, assessing anti-inflammatory activity is crucial; although the inflammatory response is essential for eliminating pathogens and damaged cells, excessive or chronic inflammation can delay tissue regeneration [19]. Anti-inflammatory activity was confirmed in studies on hydrogel patches containing *Origanum dictamnus* extract, which demonstrated that the formulation not only enabled approximately 70% wound closure (in vitro test on HaCaT cells) but also significantly reduced the levels of the proinflammatory interleukins IL-6 and TNF-α, indicating strong anti-inflammatory activity, while simultaneously increasing the level of the anti-inflammatory cytokine IL-4 [32].

Studies on antimicrobial properties have shown that hydrogels with extracts from plants of the Asteraceae and Lamiaceae families effectively inhibit the growth of various pathogens commonly associated with skin infections and wound environments. Particular attention should be paid to Gram-positive bacteria, such as *Staphylococcus aureus*, and Gram-negative bacteria, such as *Pseudomonas aeruginosa* and *Escherichia coli*, as these strains are most frequently isolated from chronic wounds, as well as to fungi of the genus *Candida*, as it has been found that burn wounds are often infected by these microorganisms [64]. An analysis of the results of studies included in this review indicates that a hydrogel containing *Rosmarinus officinalis* extract [63] and *Tussilago farfara* extract [57] exhibited antibacterial activity against strains such as *Staphylococcus aureus* and *P. aeruginosa*, while a hydrogel with *Origanum majorana* extract acted against *S. aureus* and *E. coli* [62]; a hydrogel with *Eupatorium glutinosum* extract acted against *E. coli* [54]; a hydrogel with *Ageratum conyzoides* extract acted against *S. aureus* and *E. coli* [46]; a hydrogel with *Artemisia vestita* extract acted against *E. coli*, *B. subtilis*, *S. aureus*, and *S. pyogenes* [48]; a hydrogel with *Clerodendrum glandulosum* extract acted against *E. coli*, *S. aureus* [59]; a hydrogel with *Gmelina arborea* extract acted against *B. cereus*, *E. coli*, *P. aeruginosa*, *S. aureus* [61]; and a hydrogel with *Calendula officinalis extract* acted against *S. aureus*, *P. acnes*, and *E. coli* [52], as well as against *S. aureus*, *E. coli*, *and P. aeruginosa* [51]. Furthermore, among the tested formulations, antifungal activity against *Candida albicans* was demonstrated by hydrogels containing extracts of *Artemisia vestita* [48], *Rosmarinus officinalis* [63], and *Calendula officinalis* [51]; against *Aspergillus niger*: by hydrogels containing extracts of *Artemisia vestita* [48] and *Ageratum conyzoides* [46]; and against *Aspergillus flavus*: by the hydrogel containing *Artemisia vestita* extract [48]. It can be concluded that the incorporation of plant extracts into hydrogels significantly enhances their antimicrobial activity against Gram-positive and Gram-negative bacteria as well as fungi. This highlights the promising potential and utility of such hybrid hydrogels in wound therapy, where non-antibiotic topical antimicrobial agents are preferred, as the use of topical antibiotics is associated with increased bacterial resistance and impaired healing [64].

The antioxidant activity of hydrogels containing extracts from Asteraceae and Lamiaceae plants was assessed using various methods. In one study, the antioxidant activity of alginate hydrogels containing *Artemisia annua* extract was confirmed using three methods, including DPPH, FRAP, and CUPRAC. The authors also investigated formulations containing a plant extract enriched with D-panthenol and 20% hyaluronic acid and demonstrated that the hydrogel matrix composition did not negatively affect antioxidant activity, indicating chemical compatibility with plant-derived antioxidants; at the same time, the observed higher antioxidant activity can most likely be attributed to chemical interactions between the plant constituents and the other active substances incorporated into the hydrogel [47]. Other studies using the DPPH assay demonstrated that antioxidant activity increased significantly (with maximum values observed after 24 h of incubation) as the amount of Calendula officinalis extract in the hydrogels increased, compared to the unloaded P/PVA. This study confirms the fundamental phenomenon of the DPPH-radical-scavenging method, demonstrating that a greater amount of extract, and, thus, a higher content of polyphenolic compounds, increases hydrogen-donating capacity [51]. The results of the free radical scavenging capacity of *Clerodendrum glandulosum* extract-AgNP-loaded chitosan/PEG hydrogel conducted using DPPH and ABTS methods confirmed reduction of the radicals in both assays and revealed the concentration-dependent antioxidant activity of the formulation [59]. The last of the analyzed studies demonstrated that a high-molecular-weight chitosan-based hydrogel with *Helichrysum pamphylicum* extract exhibited significant antioxidant activity, involving DPPH radical scavenging, ABTS•+ radical cation decolorization, and metal chelation, while simultaneously showing low reducing antioxidant potential [55]. It is worth noting that results described above using FRAP, DPPH, ABTS, and CUPRAC assays, derived from the articles included in this systematic review, represent in vitro chemical methods that reflect antioxidant potential under simplified conditions rather than intracellular antioxidant activity or biological efficacy in the healing process. Therefore, it would be important to confirm such biological effects in further studies, e.g., by assessing intracellular reactive oxygen species (ROS) levels in vitro using skin cell lines or in vivo studies.

#### 4.2.4. In Vivo Studies Evaluating Hydrogels Incorporating Asteraceae and Lamiaceae Plant Extracts

Table 5 summarizes the results of in vivo studies on hydrogels containing different plant extracts from the Asteraceae and Lamiaceae families. The research results show that hydrogels based on plant extracts demonstrate effectiveness through anti-inflammatory, antioxidant, and antimicrobial effects, as well as stimulation of angiogenesis, promotion of granulation tissue formation, epidermal proliferation, keratinization, collagen synthesis, and influence on the scar formation process.

**Table 5:** In vivo studies evaluating plant-extract-loaded hydrogels.

Preparation Form	Model/Wound Type	Key Results/Mechanism	Ref.
Nanoemulsions with *Achyrocline satureioides* extract–loaded CP hydrogel	rats, dorsal wound	↑ wound healing by ↑ angiogenesis, reducing inflammation (TNF-α), ↓ lipid damage, and ↑ re-epithelialization	[26]
*Arnica montana* extract-loaded SPA/PAA/CMC hydrogel patch	mice, rats	↓ paw edema thickness, anti-inflammatory (↓TNF-α, IL-1β, IL-6) activity, pain-relief effect	[27]
*Artemisia absinthium* extract-loaded CS hydrogel	rats, full-thickness burn	↑ granulation tissue formation, scar tissues developed; possible impact on the immune response within the wound	[34]
*Artemisia annua* extract-loaded SA hydrogel	rats, full-thickness skin excisions	↑ wound healing (2–3 days)	[47]
*Calendula officinalis* extract-loaded PAM hydrogel	rats, a pressure wound	↑ tissue regeneration and the healing process, ↑ collagen fibers	[49]
*Calendula officinalis* extract-AgNPs-loaded CS hydrogel	patients with type II diabetes, diabetic wound	↑ wound healing; physical protection and appropriate environmental conditions within the wound	[50]
*Calendula officinalis* extract-loaded CS hydrogel	diabetic and non-diabetic rats, full-thickness wound	↓ wound area; ↑ collagen and NAGA content	[33]
*Calendula officinalis* extract-loaded SA hydrogel	rat, incisional wound	↑ wound closure; ↑ collagen deposition, ↓ inflammation	[30]
*Clerodendrum glandulosum* extract-AgNPs-loaded chitosan-PEG hydrogel	rats, diabetic mice, circular-excision wound	anti-inflammatory (IL-6, IL-10, TNF-α) activity, ↑ wound closure rate, ↑ ECM formation	[59]
*Glechoma hederacea* extract-loaded MC hydrogel	rats, full-thickness, excisional infected wound	antibacterial (*S. aureus*) activity; full re-epithelialization, hair follicles proliferation	[60]
*Helichrysum pamphylicum* extract-loaded CS hydrogel	CAM chicken embryo	anti-inflammatory effect (HET-CAM)	[55]
*Matricaria chamomilla* extract-loaded SA/Gel hydrogel	rats, full-thickness burn wound	↑ wound healing, ↓ necrotic tissue level, ↑ angiogenesis	[56]
*Tussilago farfara* extract-loaded SA/CMC/ZnONPs hydrogel	rats, burn wound	↑ re-epithelialization and dermal formation in 14 days	[57]
*Xanthium strumarium* extract-loaded GelMA hydrogel	rats, diabetic wound	↑ wound epithelialization, skin appendage formation, and collagen deposition; ↓IL-6 and TNF-α; ↑ CD31	[58]

↑ Improves, enhances, accelerates; ↓ decreases, reduces; CP, carbopol; CS, chitosan; SPA, sodium polyacrylate; PAA, polyacrylic acid; PAM, polyacrylamide; AgNPs, silver nanoparticles; PEG, polyethylene glycol; MC, methylcellulose; SA, sodium alginate; Gel, gelatin; CMC, carboxymethylcellulose; ZnONPs, zinc oxide nanoparticles; GelMA, gelatin methacryloyl; HET-CAM, hen’s egg test on the chorioallantoic membrane; NAGA, n-acetyl glucosamine.

This part of the review summarizes scientific reports on studies conducted primarily on rodent models, as well as one study using a chick embryo model and one study involving human subjects. Topical treatments administering the hydrogel containing *A. satureioides* extract incorporated in nanoemulsion were conducted on a group of 18 rats. The research included the determination of IL-1 and TNF-α levels, myeloperoxidase (MPO) activity, and markers such as TBARS (as an indicator of lipid peroxidation and antioxidant activity), as well as histological analysis to evaluate epidermal regeneration and angiogenesis. The study results revealed that formulations containing the extract reduced inflammation and oxidative damage, as well as promoted wound healing by enhancing angiogenesis, limiting lipid damage, and improving the re-epithelialization process in lesions. Furthermore, an increased number of blood vessels and hair follicles was observed in wounds treated with the preparation compared to the control group [26]. To evaluate the pharmacological effects of an *Arnica montana* hydrogel patch on inflammation, studies were conducted using a carrageenan-induced paw edema model in mice, and RT-PCR analyses were performed to assess the levels of inflammatory markers (TNF-α, IL-1β, and IL-6). Furthermore, the pain-relieving effect of the *A. montana* patch was evaluated in rats using the hot-plate test. It was found that a hydrogel patch significantly lowered levels of inflammatory markers, limited inflammatory cell infiltration, reduced paw edema, and alleviated pain in preclinical models [27]. Chitosan-based hydrogel dressings containing 2%, 5%, or 10% *Artemisia absinthium* extract were investigated in a group of 48 rats with a burn wound model. In this study, to evaluate the wound-healing process, tissue levels of IL-1α, IL-6, IL-10, and TNF-α were measured by ELISA, and histopathological changes between days 3 and 21 were assessed. The study results indicate that changes in tissue cytokine concentrations suggest that the tested extract, when incorporated into a hydrogel, may modulate the local immune response involved in wound healing; however, the precise mechanism of this action requires further investigation. A histopathological study revealed significant changes in the appearance of the wound, with the formation of granulation and scar tissue that subsequently lightened and began to regress, particularly in the group treated with the hydrogel containing the highest concentration of *A. absinthium* extract, confirming that it promoted the healing process of burn wounds [34]. Other studies evaluated the wound-healing efficacy of sodium alginate hydrogels containing *Artemisia annua* extract, tested over 18 days on a group of 18 rats with full-thickness skin wounds. It was demonstrated that the formulations containing *A. annua* accelerated wound healing, with closure occurring 2–3 days earlier compared to the untreated group and the empty hydrogel group [47]. The efficacy of a polyacrylamide-based hydrogel containing *Calendula officinalis* extract as a wound healing agent was investigated using a rat model, involving tests such as acute dermal toxicity, assessment of cell migration, analysis of the necrotic tissue surface area, evaluation of nitric oxide activity, and histopathological assessment (including hematoxylin, eosin, and collagen evaluation), which indicates the structural or qualitative characteristics of tissues and cellular infiltrates. The tests indicated efficient collagen fiber production, improved skin repair, and no signs of dermal or systemic toxicity [49]. The healing effects of chitosan hydrogels loaded with extracts from *Calendula officinalis* were evaluated on wounds of 24 diabetic and non-diabetic rats for 13 days. Results showed that treatment with hydrogels accelerates wound contraction and enhances the deposition of extracellular matrix components (collagen and N-acetylglucosamine (precursor of hyaluronic acid) content increased compared to the control groups) [33]. The healing properties of the *Calendula officinalis* alginate hydrogel were evaluated on a group of 50 rats by measuring wound retraction rate and histological analysis of lesion tissues (inflammatory infiltrate, macrophage count, and angiogenesis) over 28 days. A significant improvement in wound closure was observed, as confirmed by histological analysis, which revealed a significant reduction in inflammatory infiltration and the number of inflammatory cells, as well as a decrease in type III collagen content and an increase in type I collagen content in the calendula-alginate hydrogel group compared to the control group. Furthermore, no significant differences in angiogenesis were found between the study groups [30]. *Clerodendrum glandulosum*-AgNPs-loaded hydrogel was evaluated using rodent models. The assessment included an examination of acute skin irritation and corrosion (symptoms such as irritation, redness, itching, and inflammation), analysis of pro- (IL-6 and TNF-α) and anti- (IL-10) inflammatory cytokines, evaluation of the oxidative status of the wound bed (the reduction of glutathione (GSH), superoxide dismutase (SOD), and catalase (CAT)); assessment of connective tissue parameters (hydroxyproline (HPR), hexosamines (HXA), and hexuronic acid (HUA) content), as well as histopathological evaluation and estimation of collagen density. It has been confirmed that the extract-based hydrogel is safe for human use and shows potential for promoting the healing of diabetic wounds by accelerating wound closure and enhancing extracellular matrix formation [59]. Wound healing activity of 2% and 5% *Glechoma hederacea*-prepared hydrogels was examined on a wounded rat model infected with *Staphylococcus aureus*. It was found that the hydrogels containing the extract caused a significant reduction in the number of colony-forming units compared to the standard Fucidin preparation, and histopathological studies demonstrated wound closure after ten days of treatment, accompanied by complete re-epithelialization and hair follicle proliferation [60]. Wound-healing efficiency of the sodium alginate/gelatin hydrogel containing chamomile extract was investigated using a full-thickness burn wound model in rats. Quantitative assessment of wound closure showed an upward trend over time, while further histopathological analysis revealed a reduced level of necrotic tissue and inflammatory cell accumulations, as well as the presence of angiogenesis, compared to the control group [56]. The efficacy of SA/CMC/ZnONPs/*T. farfara* hydrogel in healing burn wounds was evaluated in a group of 18 rats using quantitative histomorphometric analysis. Application of the preparation resulted in an increase in the thickness of granulation tissue and the epidermis, as well as the rate of angiogenesis, compared to the control group [57]. The effect of a methacrylated-gelatin-based hydrogel enriched with *Xanthium strumarium* extract on wound healing was evaluated in a rat model of diabetic wounds, assessing wound closure efficacy and performing immunohistochemical analyses of skin tissues (using TNF-α and IL-1β as markers of inflammation and anti-CD31 antibodies as markers of angiogenesis). After 21 days, compared to the control group, increased collagen deposition and improved organization of collagen fibers were observed, along with reduced levels of IL-6 and TNF-α and increased CD31 levels, indicating that the tested preparation demonstrates enhanced efficacy in the healing of diabetic wounds [58]. A chitosan-based hydrogel containing *Helichrysum pamphylicum* extract was evaluated using the HET-CAM test (hen’s egg test on the chorioallantoic membrane), an alternative in vivo animal assay to the Draize rabbit eye test. It was found that the formulation with extract exhibited a concentration-dependent anti-inflammatory effect [55]. Studies evaluating the efficacy of chitosan-based hydrogels containing silver nanoparticles and calendula extract were the only ones among those analyzed in this review to be conducted in vivo on two patients with a history of type 2 diabetes mellitus. The study demonstrated that the tested formulation creates appropriate physiological and environmental conditions within the wound, thereby stimulating the healing process and reducing the risk of infection. In both cases, a positive clinical therapeutic effect was achieved after several weeks, confirming that hydrogels promote the healing of chronic or exposed wounds and indicating their potential use as an alternative treatment method for patients with limited tissue regeneration capacity [50].

The reported research findings indicate that hydrogel formulations containing extracts from plants of the Asteraceae and Lamiaceae families may prove promising in the pursuit of improved tissue healing and regeneration.

## 5. Limitations and Future Perspectives

The approach to developing new delivery systems for natural products represents a key step toward creating effective and safe treatments for chronic wounds. It supports sustainable, eco-friendly practices in the field of biomedical materials. It is worth emphasizing that most wounds heal properly and without complications; however, special attention must be paid to appropriate management and product selection for chronic wounds, such as diabetic or venous leg ulcers. Consequently, further research is crucial to the development of modern hydrogel-plant extract composites, thereby expanding the range of dressings available for wound-healing therapy.

Undoubtedly, medicinal plants offer a promising, natural alternative for wound healing; however, their use in therapy presents certain challenges, including issues related to standardization and dosage, potential adverse effects, or contraindications, as well as establishing a clear regulatory framework and developing guidelines for the regulation and use of plant-based preparations, ensuring they meet quality, safety, and efficacy requirements.

Large-scale production of hydrogels based on plant extracts remains a challenge due to the variability of the extracts. The varying efficacy of plant-based preparations may stem from factors such as differences in plant species, cultivation conditions, harvest times, and material preparation methods; therefore, standardization and the determination of optimal dosage are crucial for minimizing variations in therapeutic outcomes. Without rigorous standardization protocols, achieving reproducibility and, consequently, obtaining regulatory approval becomes a challenge. The review indicates that many studies utilize plant extracts obtained via conventional methods, such as Soxhlet extraction or maceration. Future studies should adopt standardized guidelines for reporting extraction yield and phytochemical composition. Furthermore, it is worth considering methods that overcome certain limitations of traditional extraction techniques, such as compositional changes due to phytochemical degradation, long processing times, high solvent consumption, and the emission of potentially toxic vapors. The use of green extraction techniques, such as accelerated solvent extraction (ASE) and supercritical fluid extraction (SFE), will not only lead to the development of eco-friendly technologies but also enhance the efficacy of the final formulation.

Equally important is the development of standardized methods for incorporating plant extracts into hydrogels to ensure consistent quality and efficacy of plant-based therapies in modern medicine. The ability to scale up production will be crucial for transforming laboratory-developed solutions into commercially available topical hydrogel formulations. However, creating hydrogels for therapeutic applications in wound healing and skin regeneration that are simultaneously chemically stable, mechanically robust, stretchable, and effective remains a significant challenge. The rapid degradation of many polymer networks, particularly in electrolyte solutions, often prevents the achievement of desired therapeutic effects and, consequently, precludes the long-term in vivo application of hydrogels. Consequently, there is a continuing need to develop hydrogels with high water content and mechanical strength that are capable of maintaining their properties under demanding in vivo conditions. Understanding the mechanisms governing the balance between mechanical strength and biodegradability, including the roles of cross-linking density, polymer architecture, and degradation kinetics, will be pivotal to improving the functionality and sustainability of hydrogels in various biomedical applications. In hydrogel systems incorporating plant extracts, optimizing viscosity and spreadability is crucial to ensure topical acceptability. Moreover, extract-loaded hydrogels face additional stability challenges; for instance, plant-derived bioactive compounds may undergo oxidation or photodegradation, or interact adversely with the polymer matrix. Consequently, the manufacturing process should take into account the selection of antioxidants or stabilizers, followed by careful attention to packaging and storage conditions, including limiting exposure to light and oxygen. Large-scale production should also encompass compatibility and stability tests, including physical, chemical and microbiological stability under accelerated and real-time conditions. These tests are essential to ensure that the finished product retains its functional properties (pH, viscosity, bioactive ingredient content, absence of phase separation) under shelf life.

It is well known that many species of medicinal plants can cause adverse reactions, including allergic responses and skin irritation, or even delay the healing process. Therefore, it is crucial to deepen our understanding and conduct research into toxicity, interactions, and the safety of using plant-derived materials to gain the most comprehensive knowledge possible regarding the potential risks and contraindications associated with the use of specific plant species. Importantly, in the case of hydrogels with plant extracts for topical application, the interface between the polymer and the extract must be non-irritating and non-sensitizing. When developing biocompatible dressings, it is worth considering natural materials or hybrid matrices, which offer a non-toxic alternative to synthetic materials.

In addition to safety in use, the efficacy of hydrogels containing plant extracts with multifaceted properties (e.g., antimicrobial, anti-inflammatory, and antioxidant effects) is crucial for achieving significant clinical outcomes in wound therapy. However, achieving effective skin penetration (particularly for high-molecular-weight or hydrophilic compounds, for which the skin acts as a significant barrier) while maintaining a local effect remains a challenge. Future research should focus on formulating hydrogels that ensure both adequate skin adhesion and optimal release kinetics for active plant-derived ingredients. Such studies should consider the use of nanocarriers to enhance skin penetration and enable more precise delivery of phytochemicals. On the other hand, the use of nanocarriers faces challenges such as encapsulation efficiency and loading capacity, which are crucial for consistent therapeutic performance. Furthermore, nanoparticle-based topical hydrogels containing plant extracts and natural compounds must navigate the regulatory frameworks for nanoparticle-based systems, which unfortunately remain complex and inconsistent.

Furthermore, future research should focus on optimizing plant-based hydrogels and conducting well-designed, large-scale clinical trials to confirm their efficacy and safety, as well as to understand the long-term effects of using these advanced wound-healing products. The lack of such evidence hinders the regulatory approval process and limits the application of such formulations in clinical practice. The regulatory framework for topical herbal preparations is less precisely defined than those regulating synthetic drugs. Differences in classification (cosmetic product, medical device or drug), regulatory inconsistencies, and varying quality control standards pose a barrier to the commercialization of hydrogels containing plant extracts for wound healing and skin regeneration.

This systematic review is also subject to certain methodological limitations. Only specific databases and studies published in English were included, which may have introduced a language bias. Furthermore, the heterogeneity of study designs (various in vitro tests, different animal models) and outcome measures precluded a quantitative synthesis or meta-analysis. Consequently, the conclusions were based on a descriptive synthesis of the available data.

## 6. Conclusions

This review discusses the significance and potential application of plant-containing hydrogels in the process of skin regeneration and healing. Attention was focused on species from the Lamiaceae and Asteraceae families, and it can be concluded that they are valuable sources of natural bioactive compounds with wound-healing properties. Analysis of the chemical profiles of the extracts discussed in this review confirmed the presence of both flavonoids and phenolic acids in species belonging to both families; however, characteristic groups of compounds can be identified, such as sesquiterpene lactones in plants of the Asteraceae family, and rosmarinic acid or triterpenes (ursolic acid) in plants of the Lamiaceae family. Differences in chemical composition, and consequently in the mechanism of action and therapeutic profile, can influence how plants from these two families are utilized. The presence of compounds with antimicrobial, antioxidant, or anti-inflammatory properties in Lamiaceae species makes them particularly useful in the treatment of infected wounds accompanied by severe inflammation. Conversely, the review indicates that Asteraceae species accelerate epithelialization and stimulate fibroblast proliferation, making them especially valuable for chronic wounds with impaired tissue regeneration.

Modern hydrogel dressings, which provide a moist environment, a factor crucial for accelerated wound healing, are attracting particular interest from scientists and research teams striving to develop the ideal dressing material. This review indicates that hydrogels based on natural polymers predominated among the tested formulations, with chitosan being the most frequently selected. It was emphasized that incorporating plant extracts into hydrogels enhances their therapeutic potential by providing properties such as improved stability and the controlled release of bioactive compounds, as well as enabling infection control, immune system support, and tissue repair. The cited studies show that hydrogels enriched with plant extracts from the Lamiaceae and Asteraceae families can accelerate wound healing. It is worth noting that, despite the existence of numerous in vitro and animal studies, this review highlights a lack of clinical trials involving humans regarding plant-extract-based hydrogels.

The results discussed in this review may be useful in providing a scientific approach to the discovery and development of novel, multifunctional topical pharmaceuticals that promote regeneration processes, as the use of plant extracts in hydrogels represents a significant step forward in wound-healing technology.

## Figures and Tables

**Table 1:** Comparison of natural, synthetic and hybrid hydrogels.

Feature	Natural	Synthetic	Hybrid
Biocompatibility	high	moderate-to-high	high
Mechanical properties	moderate-to-low	very good	good to very good
Controlled drug release	moderate	good	very good
Biodegradation	fast	controlled	adjustable
Production cost	low-to-medium	medium	higher

**Table 2:** Comparison of hydrogel matrices based on wound type.

Wound Type	Preferred Matrix	Example
Dry wound	High-water-content hydrogel	CS, Gel
Wound with low to moderate exudate	Natural or hybrid hydrogel	SA, PVP, PVA
Infected wound	Hydrogel with antimicrobial properties	CS, PVP
Diabetic wound	Controlled-release, antibiofilm, anti-inflammatory, angiogenesis-promoting hybrid hydrogel	SA, CMC, PEG, PLA
Chronic ulceration	Hydrogel with high mechanical stability	PEG, PVP, CS, CBM
Burn wound	Hydrogels with cooling and pain-alleviating properties	CBM, PEG, AGR

CS, chitosan; Gel, gelatin; SA, sodium alginate; PVA, polyvinyl alcohol; PVP, polyvinylpyrrolidone; PLA, polylactic acid; CMC, carboxymethylcellulose; PEG, polyethylene glycol; CBM, carbomer; AGR, agar.

## Data Availability

No new data were created or analyzed in this study.

## Supplementary Materials

The following supporting information can be downloaded at: https://www.mdpi.com/article/10.3390/pharmaceutics18091047/s1, Figure S1: Flow diagram.

## Abbreviations

The following abbreviations are used in this manuscript:

PDGF	Platelet-derived growth factor
TGF	Transforming growth factors
TNF	Tumor necrosis factor
IL	Interleukin
FGF	Fibroblast growth factor
ECM	Extracellular matrix
IGF	Insulin-like growth factor
EGF	Epidermal growth factor
KDAF	Keratinocyte-derived autocrine factor
VEGF	Vascular endothelial growth factor
ROS	Reactive oxygen species
DPPH	2,2′-diphenyl-1-picrylhydrazyl
ABTS	2,2′-azinobis-(3-ethylbenzothiazoline-6-sulfonic acid
TBARS	Thiobarbituric acid reactive substances
FRAP	Ferric antioxidant power reduction
CUPRAC	Cupric ion reducing antioxidant capacity
SOD	Superoxide dismutase
CAT	Catalase
COX	Cyclooxygenase
LOX	Lipoxygenase
HAase	Hyaluronidase
iNOS	Inducible nitric oxide synthase
(NF)-κB	Nuclear factor kappa-light-chain-enhancer of activated B cells
AP	Activated protein
MIC	Minimum inhibitory concentration
MBC	Minimum bactericidal concentration
MFC	Minimum fungicidal concentration
AB	Alamar blue
NRU	Neutral red uptake
MTT	3-(4,5-dimethyl thiazol-2-yl)-2,5-diphenyltetrazolium bromide
HUVEC	Human umbilical vein endothelial cells
MAPK/ERK	Mitogen-activated protein kinase/extracellular signal-regulated kinase
PI3K/Akt	Phosphoinositide 3-kinase/protein kinase B
PEG	Polyethylene glycol
PVA	Polyvinyl alcohol
PLA	Polylactic acid
PVP	Polyvinylpyrrolidone
PMMA	Polymethyl methacrylate
PHEMA	Poly2-hydroxyethyl methacrylate
PU	Polyurethane
PCL	Polycaprolactone
pNIPAM	PolyN-isopropylacrylamide
FDA	Food and Drug Administration
PRISMA	Preferred Reporting Items for Systematic Reviews and Meta-Analyses
WE	Water extract
ME	Methanol extract
EE	Ethanol extract
WEE	Water–ethanol extract
DER	Drug-to-extract ratio
HAE	Heat-assisted aqueous extraction
MSE	Magnetic stirrer extraction
SAE	Shaker-assisted extraction
UAE	Ultrasound-assisted extraction
UHPLC–MS/MS	Ultra-high-performance liquid chromatography − mass spectrometry
UHPLC–DAD	Ultra-high-performance liquid chromatography with diode array detector
UFLC–PDA	Ultra-fast liquid chromatography with photodiode array detector
LC–MS/MS	Liquid chromatography–tandem mass spectrometry
GC–MS	Gas chromatography–mass spectrometry
FTIR	Fourier-transform infrared spectroscopy
TLC	Thin-layer chromatography
HPMC	Hydroxypropylmethylcellulose
SEM	Scanning electron microscope
TEM	Transmission electron microscopy
CS	Chitosan
P	Pullulan
AgNP	Silver nanoparticle
SA	Sodium alginate
Gel	Gelatin
CMC	Carboxymethylcellulose;
CV	Crystal violet
TKA	Time kill assay
SRB	Sulforhodamine B
PAA	Polyacrylic acid
PAM	Polyacrylamide
SPA	Sodium polyacrylate
MC	Methyl cellulose
ZnONPs	Zinc oxide nanoparticles
GelMA	Gelatin methacryloyl
HET-CAM	Hen’s egg test on the chorioallantoic membrane;
NAGA	N-acetyl glucosamine
MPO	Myeloperoxidase
HPR	Hydroxyproline
HXA	Hexosamines
HUA	Hexuronic acid
ASE	Accelerated solvent extraction
SFE	Supercritical fluid extraction
